# A mechanobiology-driven cell-derived ECM bioink for engineering 3D glioblastoma tumor microenvironment models

**DOI:** 10.7150/thno.137729

**Published:** 2026-07-29

**Authors:** Seohyeon An, Seoyul Jo, GeunHyung Kim

**Affiliations:** 1 Department of Precision Medicine, Sungkyunkwan University School of Medicine (SKKU-SOM), Suwon 16419, Republic of Korea.; 2 Institute of Quantum Biophysics, Department of Biophysics, Sungkyunkwan University, Suwon, Gyeonggi-do 16419, Republic of Korea.; 3 Biomedical Institute for Convergence at SKKU (BICS), Sungkyunkwan University, Suwon 16419, Republic of Korea.

**Keywords:** glioblastoma, mechanobiology, dECM bioink, 3D bioprinting, tumor microenvironment

## Abstract

Glioblastoma (GBM) is highly aggressive and difficult to treat, partly due to the lack of *in vitro* models that faithfully recapitulate its biochemical and mechanobiological microenvironment. Synthetic hydrogels lack tumor-specific cues, while animal-derived dECM suffers from batch variability, limiting standardization.

**Methods:**

Here, we describe a GBM-derived dECM bioink formulated through mechanically stimulated 3D GBM culture within GelMA/HAMA hydrogels. By controlling the matrix stiffness to match GBM tissue and applying various compressive stresses that mimic intracranial solid stress, we identified a mechanobiological activation range that maximized secretion of GBM-associated factors, including GDF15, MMP2, and MMP9.

**Results:**

The resulting bioink exhibited upregulated tumor-specific biochemical signals compared to hydrogel-only controls. Micromesh-bioprinted GBM constructs fabricated from this bioink demonstrated enhanced proliferation, invasion-related gene expression, and ECM remodeling. Co-culture with endothelial cells or fibroblasts further reconstructed stromal activation, paracrine signaling, and matrix dynamics associated with GBM progression and therapeutic resistance.

**Conclusion:**

This strategy establishes a reproducible, bioactive GBM-specific bioink platform for physiologically relevant 3D GBM modeling and GBM-on-chip applications.

## Introduction

Glioblastoma multiforme (GBM) represents the most lethal and treatment-resistant primary malignant brain tumor, with a median survival of approximately 15 months despite maximal surgical resection, radiotherapy, and temozolomide chemotherapy 1. Owing to the highly infiltrative nature, extensive intratumoral hetrogeneity, metabolic adaptability, and therapy-induced phenotypic switching, current therapeutic strategies can be severly limited 2, 3. A major challenge in GBM research is the lack of physiologically relevant, reproducible, and controllable *in vitro* models that recapitulate tumor-ECM interactions, biochemical gradients, and mechanobiological cues present in the native tumor microenvironment (TME). However, conventional 2D cultures fail to represent the spatial complexity and ECM-dependent invasion pathways of GBM, while *in vivo* xenografts are limited by species-specific matrix composition, low throughput, and challenges in controlling microenvironmental parameters 4-6.

To address these shortcomings, a broad range of 3D hydrogel platforms-such as collagen type I, hyaluronic acid, Matrigel, gelatin methacryloyl (GelMA), hyaluronic acid methacryloyl (HAMA), PEG-based hydrogels, and ECM-mimetic matrices-have been utilized to create bioengineered GBM models 7-11. Hydrogels provide controllable stiffness, controllable porosity, and meaningful biocompatibility, enabling encapsulation of tumor spheroids or patient-derived GBM cells. However, these synthetic or natural hydrogels inherently lack the diverse biochemical signals, tumor-specific ECM proteins (*e.g.*, fibronectin splice variants, tenascin-C, laminins 211/411), matricellular molecules, and growth factor reservoirs that are critical drivers of GBM proliferation, 6 maintenance, angiogenesis, and mesenchymal transition 12, 13. Notably, biochemical factors such as periostin, osteopontin, GDF15, and CXCL12 are well-documented regulators of GBM invasiveness and therapeutic resistance. However, these factors are absent or insufficiently presented in conventional hydrogel matrices 14-17. As a result, hydrogel-based GBM constructs often fail to reproduce clinically relevant features such as hypoxia-driven invasion, MMP-mediated perivascular migration, and ECM-remodeling dynamics.

To overcome the biochemical limitations of hydrogels, several studies have utilized decellularized extracellular matrix (dECM) derived from animal GBM tissues 9, 18, 19. These dECM bioinks preserve native ECM proteins, glycosaminoglycans, and tissue-specific growth factors, thereby enhancing the physiological relevance of *in vitro* tumor models 20. However, the animal-based dECM platforms struggle with significant translational challenges: (1) batch-to-batch biochemical variability owing to differences in tissue sourcing and processing, (2) absence of standardized decellularization protocols, (3) risk of incomplete removal of cellular components and immunogenic components, and (4) inadequate reproducibility in biochip integration and high-throughput applications 21, 22. These challenges underscore the need for a controllable, cell-driven, reproducible dECM platform that maintains tumor-specific biochemical factors while simultaneously supporting microengineered GBM-on-chip systems.

In this study, we introduce a GBM-derived dECM bioink formulated by culturing GBM cells in a 3D GelMA/HAMA hydrogel environment and harvesting the secreted GBM-ECM components after controlled mechanobiological stimulation. Unlike conventional dECM approaches that rely on animal tissues, the proposed bioink strategy allows precise regulation of biochemical composition, mechanotransduction stimuli (compression and stiffness), and ECM remodeling kinetics through mechanically regulated 3D GBM culture. Furthermore, by integrating the biochemical advantages of cell-derived dECM with the tunability of hydrogel-based biofabrication, this approach enables more controllable and reproducible generation of GBM-associated extracellular matrix cues than conventional tissue-derived dECM or simple cell-derived matrix models 23-25.

Importantly, GBM cells in the native environment are continuously exposed to dynamic mechanical compression resulting from intracranial pressure, tumor expansion within an enclosed skull, and solid stress generated by proliferating tumor masses. Notably, solid compressive stress within GBM is reported in the range of approximately 0.1-10 kPa, and is known to critically regulate tumor behavior 26. In addition to solid compressive stress, tumor progression and cellular behavior can also be influenced by ECM stiffness, which exerts mechanical stress on GBM cells. Recent studies have reported that ECM stiffness of GBM tissues are approximately ~10 kPa during early tumor progression and up to ~35 kPa in advanced GBM lesions, which significantly modulates tumor behavior 27, 28. However, the reported mechanical properties of GBM can vary considerably between *in vivo* and *ex vivo* or *in vitro* measurements because of differences in measurement methods and physiological conditions 29. Despite these differences, biomechanical cues are recognized as the regulators of GBM progression, emphasizing the importance of developing physiologically relevant *in vitro* GBM models with improved predictive reliability.

Previous studies have demonstrated that compressive loading regulates GBM progression by altering cellular mechanotransduction, invasion, and gene expression 30-33. Through activation of integrin–FAK, MAPK, NF-κB, and YAP/TAZ pathways, the mechanical stimulations have been reported to influence the GBM proliferation, metabolic rewiring, mesenchymal transition, and ECM remodeling 34. However, these studies have primarily focused on characterizing the biological responses to compression, whereas the utilization of mechanically remodeled GBM-derived ECM as a bioactive material for biofabrication has been rarely explored. Therefore, we hypothesized that applying controlled compressive stress during 3D culture would upregulate key tumor-derived biochemical factors, such as GDF15, MMP2, and MMP9, that play essential roles in GBM invasiveness and ECM degradation.

By controlling various compressive stress levels during 3D GBM culture, we have identified compressive conditions in which the secretion of tumor-associated remodeling enzymes and cytokines is maximized; therefore, ECM-enriched bioinks with strong tumor-mimetic bioactivity are obtained. These biochemical factors were analyzed to determine an appropriate pressure range for the GBM-derived ECM harvesting, enabling the formulation of a bioink with reproducibility and enhanced tumor-relevant signaling.

Furthermore, mimicking the native mechanical stiffness of brain and GBM tissues can be essential for reconstructing biologically relevant TME models. Normal brain tissue exhibits a relatively low stiffness (~0.2-1.5 kPa), whereas GBM regions are moderately stiffer (~1-35 kPa) 35. Therefore, hydrogels modified within this physiologically relevant range can regulate mechanotransduction pathways, including YAP/TAZ and PI3K/AKT, more accurately and control GBM proliferation, invasion, stemness, and drug response. Consequently, stiffness-matched matrices better reproduce *in vivo*-like tumor behavior and improve the predictive reliability of *in vitro* models. Based on this rationale, we investigated various mechanical stiffnesses of GBM-laden hydrogels by modulating the GelMA concentration.

After decellularization, the harvested GBM-derived dECM was incorporated within the composite bioink as a dispersed phase. Through careful selection of dECM concentration where cytocompatibility and rheological stability were established, micromesh-structured GBM constructs were successfully fabricated. The biological performance of the biocomposite constructs was compared with conventional GelMA/HAMA-only constructs to assess biological performance, including GBM proliferation, invasion-associated gene expression, and MMP activity. Notably, the biocomposite constructs significantly enhanced tumor aggressiveness and invasion-related phenotypes, demonstrating their enhanced ability to recapitulate functional GBM TME features.

GBM progression is regulated not only by tumor-specific reprogramming but also by dynamic interactions with the surrounding tumor microenvironment. Although the brain parenchyma lacks classical fibroblasts, GBM reprograms resident stromal populations into mesenchymal-like, matrix-remodeling phenotypes that functionally resemble fibroblast-like stromal cells in other solid tumors. Accordingly, fibroblasts have been used as a surrogate model for activated, ECM-remodeling stromal components, while endothelial cells can regulate the perivascular niche and angiocrine signaling. In this context, to reconstruct a more physiologically relevant tumor microenvironment, GBM constructs were co-cultured with either endothelial cells (EA.hy926) or fibroblasts (human dermal fibroblasts), allowing the recapitulation of stromal activation, paracrine crosstalk, and dynamic matrix remodeling that drive GBM invasion and therapeutic resistance.

Accordingly, this work can establish an alternative strategy for obtaining GBM-mimetic ECM environments guided by mechanotransduction-driven biochemical outputs. Our approach provides a controllable, reproducible, and bioactive GBM-specific dECM bioink, overcoming the limitations of conventional synthetic hydrogels and less controllable animal-derived dECM. This proof-of-concept platform can be used for mechanobiology studies, therapeutic evaluation, and GBM-on-chip applications.

## Materials and Methods

### Materials and cell culture

The human glioblastoma cell line SF295 was kindly provided by Prof. Hoon Kim (College of Pharmacy, Sungkyunkwan University, Suwon, Republic of Korea). SF295 cells were cultured in RPMI-1640 medium (Servicebio, China, Cat No. G4535) supplemented with 10% fetal bovine serum (FBS; Sigma Aldrich, USA, Cat No. F1283) and 1% penicillin/streptomycin (PS; Capricorn Scientific, Germany, Cat No. PS-B). Human endothelial cells (EA.hy926; ATCC, USA) and human dermal fibroblasts (HDF; ATCC, USA) were maintained in high-glucose DMEM (Welgene, Korea, Cat No. LM 001-05) containing 10% FBS and 1% PS. All cells were cultured at 37 °C in a humidified incubator with 5% CO₂, and media were refreshed every 2–3 days.

GelMA and HAMA were synthesized via methacrylation as previously reported 36-38. Briefly, gelatin (10 w/v%; MP Biomedicals, USA, Cat No. 901771) in phosphate-buffered saline (PBS) was reacted with methacrylic anhydride (Sigma Aldrich, USA, Cat No. 760-93-0) at 50 °C, dialyzed (3.5 kDa MWCO; Thermo Fisher Scientific, USA, Cat No. 086705B), and lyophilized. HAMA was prepared by reacting hyaluronic acid (1 w/v%; Sigma Aldrich, USA, Cat No. 943096) with methacrylic anhydride at 4 °C (pH 8), followed by dialysis and lyophilization.

### Compression protocol and hydrogel optimization

A preliminary study was performed to determine the maximum cytocompatible compressive stress. SF295 cells were seeded as monolayers (1 × 10⁴ cells cm⁻²), cultured for 3 days, and then exposed to static compression for 6 h using a previously established loading system 30,32. To achieve uniform load distribution, a 2 w/v% low-melting agarose cushion (BioShop, Canada, Cat No. AGA001) was placed over the cell monolayer before calibrated weights were applied to generate compressive stresses of 0, 0.05, 0.1, and 0.15 kPa. Unloaded cells served as the control group.

To determine an appropriate hydrogel formulation, GelMA precursor solutions (3–10 w/v%) were prepared in PBS supplemented with HAMA (0.25 w/v%) and lithium phenyl-2,4,6-trimethylbenzoylphosphinate (0.05 w/v%, LAP; Sigma Aldrich, USA, Cat No. 900889). SF295 cells were encapsulated at 1 × 10⁷ cells mL⁻¹, cast into cylindrical molds (5 mm diameter × 3 mm height), and crosslinked by UV irradiation (0.5–1.5 J cm⁻², UV LED Light Curing; TAORLAB, China). Compressive modulus was measured under wet conditions using a universal testing machine (SurTA; Chemilab, Republic of Korea) at a compressive speed of 0.1 mm/s and calculated from the linear region of stress–strain curves.

### Inhibition of mechanotransduction-associated signaling

To investigate the involvement of mechanotransduction-associated signaling, *Piezo1* and *YAP/TAZ* inhibition experiments were performed using GsMTx4 (MedChemExpress, USA, Cat No. HY-P1410) and verteporfin (MedChemExpress, USA, Cat No. HY-B0146), respectively. Cell-laden constructs were fabricated, photocrosslinked, and cultured prior to inhibitor treatment. GsMTx4 was added to the culture medium at a final concentration of 5 μM to inhibit *Piezo1*-associated mechanosensitive ion channel activity 39, 40. Verteporfin was dissolved in DMSO and added to the culture medium at a final concentration of 0.1 μM to inhibit *YAP/TAZ*-associated transcriptional signaling 41.

### Quantification of collagen and sulfated glycosaminoglycans

Collagen and sulfated glycosaminoglycan (sGAG) contents were quantified before and after decellularization. Lyophilized samples were weighed before analysis, and three independently prepared samples were included for each group.

Collagen was quantified using the Sircol Soluble Collagen Assay Kit (Biocolor Ltd., UK, Cat No. S1000) following the manufacturer's protocol. Collagen was extracted from the lyophilized samples with an acid–pepsin extraction solution, reacted with Sirius Red dye, and quantified by measuring absorbance at 555–556 nm using a microplate reader. The collagen content was calculated from the standard curve provided with the kit and normalized to the dry sample weight.

The sGAG content was quantified using the Blyscan Sulfated Glycosaminoglycan Assay Kit (Biocolor Ltd., UK, Cat No. B1000) according to the manufacturer's instructions. Lyophilized samples were digested with papain extraction reagent, reacted with 1,9-dimethylmethylene blue dye reagent, and quantified by measuring absorbance at 656 nm. The sGAG concentration was calculated using a chondroitin-4-sulfate standard curve and normalized to the sample dry weight.

### Preparation of GBM-derived dECM and composite bioinks

Cell-derived dECM was generated from SF295-laden GelMA/HAMA hydrogels (1 × 10⁷ cells/mL). After stabilization for 3 days, the constructs were exposed to compressive loading (0.1 kPa, 6 h) and cultured for an additional 7 days to allow ECM deposition. The constructs were then decellularized by four freeze–thaw cycles (-80°C to 25°C), followed by treatment with 0.05% sodium dodecyl sulfate for 30 min and 0.5% Triton X-100 for 24 h under gentle agitation. After three washes PBS and distilled water (DW) (10 min each), the cell-derived dECM was then lyophilized and cryomilled into fine powder for bioink preparation. Non-compressed cell-derived dECM was generated using the same protocol without compression. GelMA/HAMA powder was prepared in the same manner but without cells.

Composite bioinks were formulated by incorporating GelMA/HAMA, non-compressed cell-derived dECM, or compressed cell-derived dECM powders (50–150 mg mL⁻¹) into a GelMA (1 w/v%) matrix hydrogel.

### Rheological characterization

Rheological properties were measured using a rotational rheometer (Discovery Core Rheometer, TA Instruments, USA) equipped with a 40 mm parallel plate (1 mm gap). Frequency sweeps (1–100 Hz, 1% strain, 25 °C), temperature sweeps (1 Hz, 1% strain, 1 °C min⁻¹), and time sweeps were conducted (1% strain, 1 Hz, 25 °C), with UV exposure (20 s) applied during the time sweep.

### 3D bioprinting

For 3D bioprinting, an extrusion-based printing system (DTR3-2210 T-SG; DASA Robot, Republic of Korea) equipped with a dispenser (AD-3000C; Ugin-tech, Republic of Korea) was used, following previously reported methods 42. Briefly, the following printing conditions were fixed: barrel temperature (25 °C), nozzle moving speed (20 mm s⁻¹), nozzle diameter (500 μm), and nozzle length (2 cm). Mesh constructs were printed by varying pneumatic pressure and UV intensity, and strut diameters were quantified using an optical microscope (CKX41; Olympus, Japan) and ImageJ software (National Institutes of Health, Bethesda, MD, USA) with the built-in measurement tools. No additional plugins or extensions were used. As a result, the optimized pneumatic pressure for each group was set to 100 kPa for C-dECM, 80 kPa for NC-dECM, and 60 kPa for the powder group, while the UV intensity was maintained at 50 mW cm⁻² for all groups. To further maintain stable 3D structures, additional UV light exposure (UV dosage = 50 mJ cm⁻²) was applied post fabrication. Print fidelity was assessed using printability (P_r_) analysis (P_r_ = πL^2^/4A), where L is the perimeter, and A is the area of the pore, and bridging tests 43, 44.

### *In vitro* cellular activities

All cell constructs were cultured in six-well culture plates using RPMI1640 medium supplemented with 10% FBS and 1% PS at 37 °C in a humidified atmosphere containing 5% CO₂. The culture medium was refreshed every 2-3 days.

Cell viability within the 3D bioprinted constructs was assessed using a Live/Dead viability assay. The samples were incubated with 0.15 μM calcein AM (Invitrogen, USA, Cat No. C1429) and 2 μM ethidium homodimer-1 (Sigma Aldrich, USA, Cat No. 46043) for 1 h at 37 °C. Live cells were identified by green fluorescence, whereas dead cells were identified by red fluorescence. Fluorescence images were obtained using a confocal laser scanning microscope (LSM 700; Carl Zeiss, Germany), and cell viability was quantified with the built-in measurement tools in ImageJ software. No additional plugins or extensions were used.

Cell proliferation of GBM cells encapsulated within the 3D hydrogel was evaluated using a Cell Counting Kit-8 assay (CCK-8; Dojindo Laboratories, Kumamoto, Japan) according to the manufacturer’s protocol. Briefly, the constructs were incubated in culture medium containing 10 v/v% CCK-8 reagent for 2 h at 37 °C. The supernatant was then transferred to a 96-well plate, and absorbance was measured at 450 nm using a microplate reader.

Cell morphology, including cell nuclei and actin filaments (F-actin), was visualized by DAPI/phalloidin staining. The constructs were fixed with 3.8% paraformaldehyde (Sigma Aldrich, St. Louis, USA, Cat No. 252549) for 1 h at 37°C and permeabilized with 0.1% Triton X-100 for 20 min at 37 °C. The cells within the bioconstructs were then stained with 4′,6-diamidino-2-phenylindole (DAPI; diluted 1:100 in PBS; Invitrogen, USA, Cat No. D35171) and Alexa Fluor 488 phalloidin (diluted 1:100 in PBS; Invitrogen, USA, Cat No. A12379).

### Immunofluorescence analysis

To further evaluate cellular activities, immunofluorescent staining of Ki67, Piezo1, vimentin, HIF-1α, EGFR, and CD31 was performed. Briefly, the cells within the bioconstructs were fixated with 3.7% paraformaldehyde for 1 h and permeabilized with 2% Triton X-100 for 2 h. After blocking with 2% bovine serum albumin (BSA; Sigma Aldrich, USA, Cat No. A9647) for 2 h at 37 °C, the samples were incubated overnight at 4 °C with primary antibodies against Ki67 (1:200 in PBS; ABclonal, Republic of Korea, Cat No. A20018), Piezo1 (1:200 in PBS; Alomone Labs, Israel, Cat No. APC-087), vimentin (1:200 in PBS; Abcam, UK. Cat No. ab137321), HIF-1α (1:200 in PBS; Novus Biologicals, USA, Cat No. NB100-449), EGFR (1:200 in PBS, ABclonal, Republic of Korea, Cat No. A23764), and CD31 (1:100 in PBS, Invitrogen, USA, Cat No. 14-0311-82). After washing with PBS, the constructs were incubated with Alexa Fluor–conjugated secondary antibodies (1:500 in PBS; Abcam, UK, Cat No. A-11032 and A-11034) for 90 min. Nuclei were counterstained with DAPI (1:100 in PBS) for 30 min. Fluorescence images were acquired using a confocal laser scanning microscope, and ImageJ software was used for quantitatively analysis by calculating the percentage of fluorescence-positive area from confocal images. No additional plugins or extensions were used.

### Real-time polymerase chain reaction (RT-qPCR)

To quantify gene expression levels related to mechanotransduction, extracellular matrix remodeling, and stromal signaling, quantitative real-time polymerase chain reaction (RT-qPCR) was performed. Initially, total RNA was isolated from the samples using TRI Reagent (Sigma Aldrich, USA, Cat No. T9424) according to the manufacturer’s instructions. The concentration and purity of the extracted RNA were assessed using a spectrophotometer (FLX800T; BioTek, USA). Subsequently, complementary DNA (cDNA) was synthesized from RNase-free DNase-treated total RNA using a ReverTra Ace qPCR RT Master Mix (Toyobo, Japan, Cat No. FSQ-201). Quantitative real-time PCR was conducted using a StepOnePlus Real-Time PCR System (Applied Biosystems, USA) with gene-specific primers. The expression levels of target genes were normalized to the housekeeping gene GAPDH. Relative gene expression levels were calculated using the comparative Ct (2^-ΔΔCt) method.

PCR products collected after 30 cycles of RT-qPCR were stained with LoadingSTAR (Dyne Bio, Republic of Korea, Cat No. A760) and resolved on a 1.2% agarose gel by electrophoresis. The gels were imaged using a ChemiDoc XRS+ gel documentation system (Bio-Rad Laboratories, Hercules, CA, USA). The corresponding uncropped and unprocessed raw images are provided in Supplementary information. The sequences of gene-specific primers used in this study are listed in Supplementary Table S1.

### Drug treatment assay

Cell-laden constructs were fabricated on day 0 and cultured for 7 days. Subsequently, the culture medium was replaced with fresh medium containing 500 μM temozolomide (TMZ; Sigma-Aldrich, USA, Cat. No. 5.00609), and the constructs were incubated for up to 72 h 45. Cell metabolic activity was evaluated using the CCK assay at 24, 48, and 72 h after TMZ treatment. Cell viability was assessed using Live/Dead staining before (day 7) and after TMZ treatment (day 10), while drug response-associated gene expression was analyzed by RT-qPCR on day 10.

### Co-culture Experiments

GBM constructs (powder, NC-dECM, and C-dECM) were co-cultured with HDFs (5 × 10⁶ cells/mL) or EA.hy926 cells (1 × 10⁷ cells/mL) together with SF295 cells (1 × 10⁷ cells/mL). After co-culture, immunofluorescence staining and RT-qPCR were carried out to assess CAF-like activation and EndMT-associated transcriptional changes. The culture medium was prepared by mixing RPMI 1640 and high-glucose DMEM in a 1:1 ratio, supplemented with 10% FBS and 1% PS.

Additionally, the C-dECM-P bioconstruct was fabricated using an extrusion-based printing system. Briefly, the C-dECM bioink was used to position two distinct regions: a central tumor region containing SF295 cells (1 × 10⁷ cells/mL) and a peripheral endothelial region containing EA.hy926 cells (1 × 10⁷ cells/mL). The bioconstructs were evaluated using immunofluorescent staining of EGFR and CD31.

### Diffusion assay

To compare molecular diffusion between bulk and mesh constructs, a rhodamine B-based diffusion assay was performed. Rhodamine B was mixed with the C-CB bioink at a final concentration of 0.1 mM 46. Bulk and mesh constructs with comparable material volumes were fabricated under identical conditions.

After photocrosslinking, each construct was immersed in DPBS, and rhodamine B diffusion from the constructs into the surrounding solution was monitored for 30 min. Representative images were acquired at 0 and 30 min. The absorbance of the surrounding solution was measured at 554 nm at 0, 5, 10, 15, 20, 25, and 30 min using a microplate reader 47.

### Statistical analysis

All quantitative data are presented as mean ± standard deviation (SD). Statistical analyses were performed using SPSS software (SPSS Inc., Chicago, IL, USA). Comparisons between two groups were conducted using Student’s t-test, while comparisons among multiple groups were performed using one-way analysis of variance (ANOVA) followed by Tukey’s honestly significant difference (HSD) post hoc test. Statistical significance was defined as p* < 0.05, p** < 0.01, and p*** < 0.001.

## Results and Discussion

**Figure 1:**
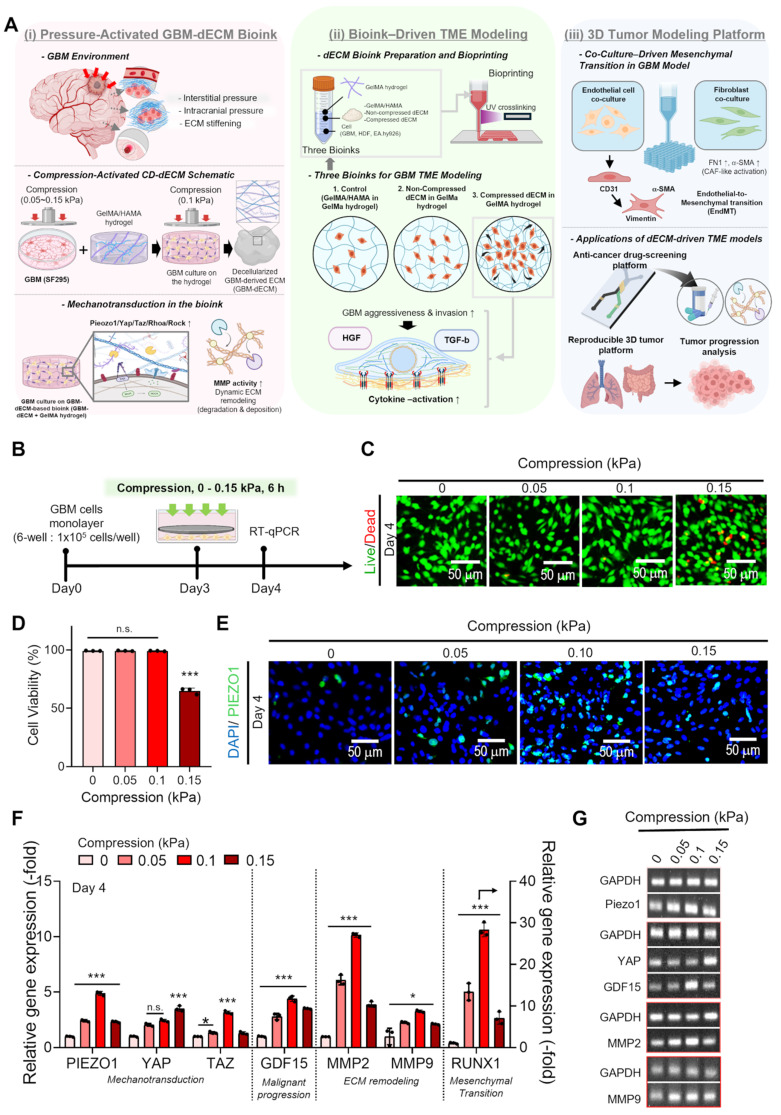
** Compression-activated generation and mechanobiological validation of a GBM-derived dECM bioink. (A)** Schematic illustration of the mechanobiology-driven GBM-dECM bioink platform. (i, ii) GBM (SF295) cells encapsulated in GelMA/HAMA hydrogels were subjected to graded compressive stresses (0–0.15 kPa) to induce mechanotransduction-mediated ECM remodeling, followed by decellularization and subsequent incorporation of the compression-conditioned dECM into a GelMA matrix for UV-assisted 3D mesh bioprinting. (iii) Application of the fabricated constructs as a 3D tumor microenvironment (TME) model. **(B)** Experimental procedure for applying controlled compressive stresses to GBM monolayers. **(C)** Live (green)/Dead (red) staining images showing maintained cell viability at ≤0.1 kPa and increased cytotoxicity at 0.15 kPa. Scale bars, 50 μm. **(D)** Quantitative analysis of cell viability (n = 3) identifying 0.1 kPa as the optimal cytocompatible mechanical activation window. **(E)** DAPI (blue)/Piezo1 (green) immunofluorescence images demonstrating enhanced mechanosensitive signaling at 0.1 kPa. (f) RT-qPCR analysis (n = 3) of mechanotransduction- and malignancy-associated genes (*Piezo1, YAP/TAZ, GDF15, MMP2, MMP9,* and* RUNX1*), showing maximal transcriptional activation under intermediate compression. **(G)** Representative agarose gel electrophoresis. One-way ANOVA followed by Tukey's HSD post-hoc test was used for multiple comparisons.

Figure 1A presents the overall framework of the compression-activated, dECM-driven GBM bioink platform for reconstructing a physiologically relevant 3D TME. As illustrated, the native GBM environment is characterized by elevated interstitial/intracranial pressure and ECM stiffening, which were recapitulated *in vitro* by applying controlled compressive stimuli (0.05–0.15 kPa) to GBM (SF295)–laden GelMA/HAMA hydrogels. This mechanical conditioning enhanced tumor-specific matrix remodeling and biochemical cue deposition. After decellularization, the compression-activated GBM-derived dECM was incorporated as a dispersed bioactive phase within a GelMA hydrogel matrix to fabricate printable composite bioinks (Figure 1A-(i)).

Three bioink formulations [(i) control (GelMA/HAMA in GelMA hydrogel), (ii) non-compressed dECM in GelMA hydrogel, and (iii) compressed dECM in GelMA hydrogel] were prepared and fabricated into 3D mesh constructs *via* UV-assisted bioprinting (Figure 1A-(ii)). The compressed dECM bioink promoted mechanotransductive activation (*e.g.*, *PIEZO1/YAP/TAZ/RhoA/ROCK*), enhanced *MMP* activity and dynamic ECM remodeling, and induced cytokine-associated signaling (*HGF, TGF-β*), resulting in increased GBM aggressiveness and invasion. Furthermore, co-culture with endothelial cells and fibroblasts within the 3D bioprinted constructs supported endothelial-to-mesenchymal transition (EndMT) and CAF-like activation, enabling a reproducible platform for anti-cancer drug screening and tumor progression analysis (Figure 1A-(iii)).

### Development of a compression-activated GBM-dECM bioink

To define the pressure threshold that activates GBM mechanobiological responses without inducing excessive cytotoxicity, we applied a range of compressive stresses (0–0.15 kPa) to GBM monolayers (1 × 10^5^ cells/well) for 6 h, as depicted in Figure 1B. Live (green)/Dead (red) staining after compression (Figure 1C) revealed that cell viability remained high (>95%) and comparable to the non-compressed control between 0 and 0.1 kPa, whereas a noticeable increase in dead cells appeared at 0.15 kPa. Quantitative cell viability (Figure 1D) confirmed this trend, showing a sharp decrease in survival only at 0.15 kPa, indicating that this level of compression exceeds the physiological tolerance of GBM cells. In addition, immunofluorescence staining for DAPI (blue)/Piezo1 (green) showed that intermediate compression, particularly 0.1 kPa, elicited the strongest mechanobiological response (Figure 1E). Consistently, RT-qPCR and electrophoresis results revealed maximal upregulation of mechanotransduction regulators (*Piezo1* and *YAP/TAZ*), the malignant progression marker *GDF15*, ECM-remodeling enzymes (*MMP2/MMP9*), and *RUNX1* at 0.1 kPa (Figure 1F-G). Based on the results, this selective enhancement at 0.1 kPa may be attributed to an appropriate mechanical activation window in which cytoskeletal tension, integrin clustering, and nuclear deformation can be sufficient to stimulate mechanosensitive transcriptional pathways without inducing cell damage or mechanotransductive shutdown 48, 49.

GelMA–HAMA formulations containing 3–10 w/v% GelMA and a fixed HAMA concentration (0.25 w/v%) were evaluated to identify a suitable hydrogel matrix for bioink fabrication 50,51. GelMA served as the primary matrix because it contains RGD-mediated adhesion motifs and MMP-degradable sequences that support interactions between GBM cells and the surrounding matrix 52. HAMA was included to reproduce the hyaluronic acid–rich environment of the brain tissue, where HA-mediated signaling through CD44 and RHAMM contributes to GBM cell migration, stemness, and therapeutic resistance 53, 54.

**Figure 2:**
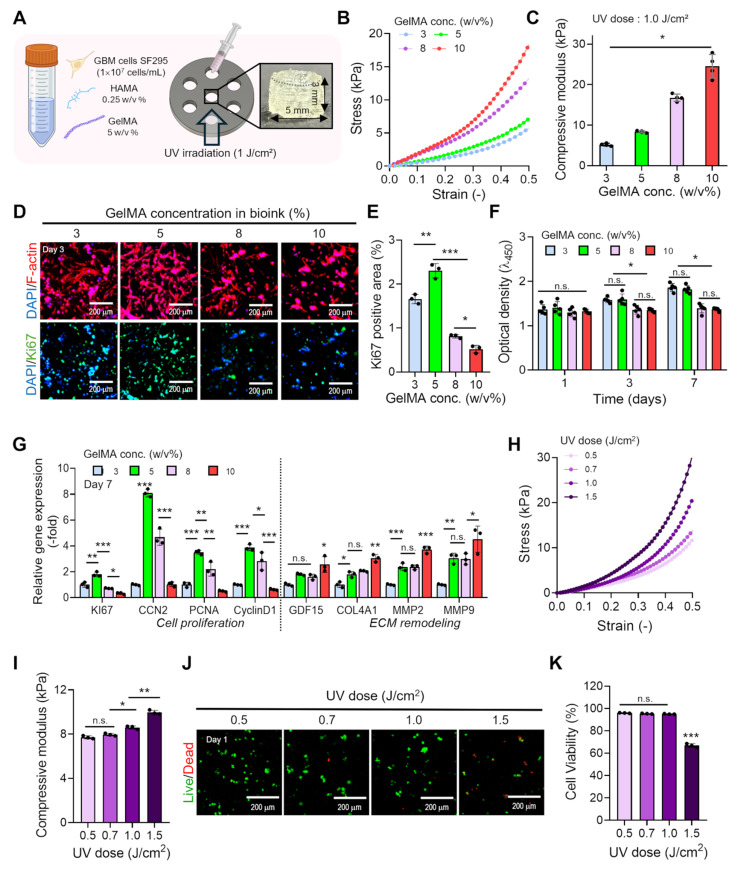
** Selection of a stiffness-matched GelMA/HAMA hydrogel for cytocompatible and tumor-permissive GBM constructs. (A)** Fabrication scheme of GBM-laden GelMA/HAMA hydrogels with tunable GelMA concentrations. **(B,C)** Representative compressive stress–strain curves and corresponding compressive modulus (n = 4) demonstrating stiffness-dependent mechanical tuning (≈ 5–25 kPa) with increasing GelMA content. **(D)** DAPI/F-actin (red) and Ki67 (green) immunofluorescence images and **(E)** quantitative analysis of Ki67⁺ area (n = 3), revealing proliferation changes across GelMA concentrations. **(F)** Cell proliferation (n = 5) confirming stiffness-dependent modulation of GBM growth. **(G)** RT-qPCR analysis of genes associated with cell proliferation and ECM remodeling, indicating enhanced stiffness-driven aggressiveness-related transcriptional responses. **(H)** UV dose–dependent stress–strain curves, **(I)** corresponding compressive modulus (n = 3), **(J)** Live/Dead staining images, and **(K)** cell viability of GBM-laden GelMA (5 w/v%)/HAMA constructs (n = 3). One-way ANOVA followed by Tukey's HSD post-hoc test was used for multiple comparisons.

To improve *in vivo*-like tumor behavior and predictive accuracy, GBM TME models require stiffness-matched hydrogels (0.1-10 kPa) to appropriately regulate *YAP/TAZ* and *PI3K/AKT* signaling. To obtain hydrogels with various stiffness, we fabricated GBM-laden constructs by mixing SF295 GBM cells (1 × 10⁷ cells/mL) with various concentrations of GelMA (3~10 w/v%) and fixed HAMA concentration (0.25 w/v%). Subsequently, the bioink solution was injected into a cylindrical mold (diameter, 5 mm; height, 3 mm) and crosslinked by UV irradiation (1 J cm^-^²) (Figure 2A).

After crosslinking, compressive stress-strain analysis showed that the compressive modulus of the cell constructs increased proportionally with GelMA concentration, ranging from approximately 5 to 25 kPa across the 3–10 w/v% GelMA concentrations (Figure 2B-C). To investigate the influence of stiffness on GBM cellular behavior, we evaluated cell proliferation and the expression of invasion- and mechanotransduction-related genes, including *GDF15* and *MMP2/9*. Ki67 staining (Figure 2D-E), MTT assays (Figure 2F), and proliferation-related genes (Figure 2G) revealed that GBM proliferation significantly declined when GelMA content exceeded 5 w/v%. In contrast, cancer aggressiveness-related gene expression (*GDF15, COL4, MMP2, and MMP9*) steadily increased as modulus increased (Figure 2G).

This inverse trend suggests that, although overly stiff matrices (>10 kPa) limit cell spreading, metabolic activity, and nutrient/oxygen transport, GBM cells activate a stiffness-adaptive transcriptional program. Specifically, increased *GDF15* indicates mechanosensitive stress signaling; elevated *COL4* reflects ECM deposition and niche reconstruction; and higher *MMP2/MMP9* expression signifies enhanced ECM degradation and invasiveness. Collectively, these changes can imply that GBM cells subjected to higher stiffness transition toward a more aggressive phenotype characterized by stress adaptation, ECM remodeling, invasive capacity, stemness maintenance, and potential therapy resistance. Therefore, we selected a HAMA (0.25 w/v%)/GelMA (5 w/v%) formulation that supports both tumor activity and cell viability. Notably, this composition yields a stiffness (~8 kPa) comparable to that of native GBM tissue, further justifying its use for subsequent bioink development 45, 55.

We further evaluated the effects of UV exposure (0.5–1.5 J cm^-^²) on the GBM-laden constructs. The compressive modulus increased with increasing UV dose, reflecting enhanced crosslinking density (Figure 2H-I). However, cell viability markedly decreased when the UV dose exceeded 1 J cm^-^², likely due to photocytotoxicity induced by excessive radical generation (Figure 2J-K). These results can indicate that UV exposure must be carefully selected to balance hydrogel mechanical stability with laden GBM survival.

**Figure 3:**
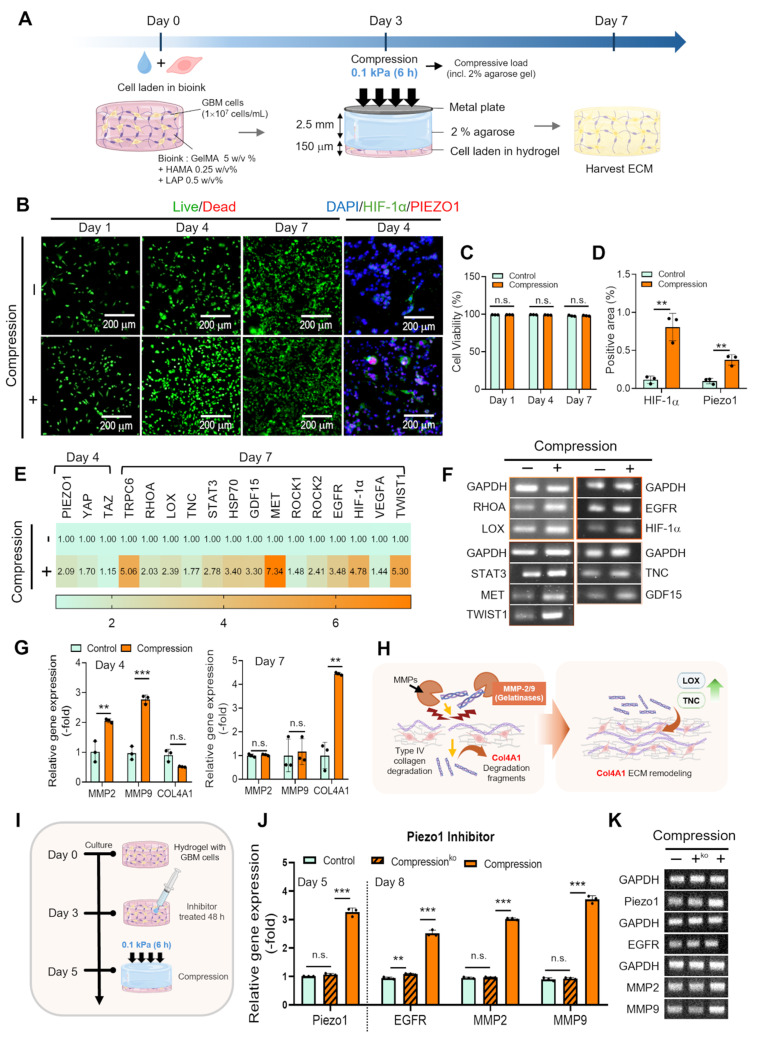
** Compression-induced mechanotransduction and temporal ECM remodeling in 3D GBM-laden hydrogels. (A)** Schematic of the compression platform. GBM-laden GelMA/HAMA 3D constructs were pre-cultured, overlaid with agarose/metal plate to ensure uniform load distribution, and subjected to 0.1 kPa compression for 6 h to activate mechanotransduction prior to ECM harvesting. **(B)** Live/Dead staining at days 1, 4, and 7 and DAPI/HIF-1α (green)/PIEZO1 (red) immunofluorescence images at day 4. **(C)** Cell viability (n = 3) and **(D)** corresponding HIF-1α⁺ and PIEZO1⁺ areas (n = 3). **(E)** Heat map of mechanotransduction-, hypoxia-, invasion-, and ECM-related gene expression (n = 3) after compression.** (F)** Representative agarose gel electrophoresis. **(G)** Relative expression of ECM remodeling markers (MMP2, MMP9, and COL4A1) at days 4 and 7 (n = 3). **(H)** Schematic of biphasic ECM remodeling showing early MMP-mediated matrix degradation and microtrack formation followed by collagen and vascular ECM deposition. **(I)** Experimental timeline for inhibition of *Piezo1*-associated mechanosensitive signaling. **(J)** Relative expression (n = 3) of Piezo1 on day 5 and EGFR, MMP2, and MMP9 on day 8 in the Control, Compression^ko^, and Compression groups.** (K)** Representative agarose gel electrophoresis images corresponding to *Piezo1*, *EGFR*, *MMP2*, and *MMP9* expression. Student's t-test was used for two-group comparisons. One-way ANOVA followed by Tukey's HSD post-hoc test was used for multiple comparisons.

Figure 3A depicts the mechanical compression platform applying 0.1 kPa, a condition obtained using monolayer GBM cultures, on GBM-laden GelMA/HAMA hydrogel (1 × 10⁷ cells/mL in 5 w/v% GelMA, 0.25 w/v % HAMA, and 0.05 w/v% LAP). Bulk constructs were used for the compression experiments because the bioprinted mesh architecture, consisting of intersecting filaments, can generate non-uniform stress under external compression. Indeed, cell viability in the compressed mesh constructs was unevenly distributed between different regions after compression (Figure S1A). To determine whether the constructs exhibit such mechanotransduction responses, compressed (+) and non-compressed (-) GBM-laden hydrogels were compared using Live/Dead images at 1, 4 and 7 days and DAPI (blue)/HIF-1α (green)/Piezo1 (red) analysis at 4 days (Figure 3B-D). Live/Dead staining showed that both groups maintained high GBM viability (over 90%), confirming that the applied compression (0.1 kPa) was a non-destructive biomechanical stimulus that does not induce cytotoxicity, whereas increased cell death was observed at 0.15 kPa (Figure 3 and Figure S1B). In contrast, HIF-1α and Piezo1 signals were noticeably elevated in the compressed group (Figure 3D). The increase in HIF-1α can indicate that the compression generates a confined or pseudo-hypoxic microenvironment, which is well known to enhance GBM metabolic adaptation and support stem-like behavior 56. Furthermore, the higher expression of Piezo1 in compression suggests activation of a mechanosensing program that promotes Ca²⁺ influx and initiates downstream cytoskeletal and transcriptional changes associated with tumor aggressiveness 57.

RT-qPCR analysis further confirmed these observations across a broad gene panel aligned with the signaling pathways depicted in Figure S2A. The overall expression profiles are presented in the heat map (Figure 3E and Figure S2B), and representative electrophoresis results are shown in Figure 3F. Compression increased the expression of genes involved in several biological processes. Mechanotransduction-related genes, including *PIEZO1, TRPV4, RHOA, ROCK1/2,* and *ACTA2*—were consistently upregulated. Genes associated with hypoxia and metabolic stress (*HIF-1α*, *BNIP3*, and *VEGFA)*, invasion- and stemness-associated regulators (*STAT3*, *TAZ*,* MET*, *TWIST1*, and *VIMENTIN)*, and ECM remodeling (*MMP2*, *MMP9*, *COL4A1*, and *LOX)*—also showed higher expression than those in the non-compressed group. Together, these transcriptional changes indicate that mechanical compression broadly influences pathways involved in GBM progression.

To examine changes in ECM remodeling over time, the expression of *MMP2*, *MMP9*, and *COL4* was analyzed on days 4 and 7 (Figure 3G). On day 4, compressed cell-constructs showed higher expression of *MMP2* and *MMP9* expression than the non-compressed group, indicating enhanced proteolytic activity during the early stage of culture. Elevated expression of these enzymes is also consistent with the formation of microtracks that facilitate tumor cell spreading and invasion. By day 7, *COL4* expression increased, suggesting a transition from matrix degradation to matrix deposition. This temporal change is consistent with the biphasic ECM remodeling behavior observed in native GBM, where early proteolytic activity is followed by basement membrane deposition that supports the development of a tumor-associated extracellular matrix (Figure 3H) 58-60.

To investigate the involvement of *Piezo1*- and *YAP/TAZ*-associated mechanotransduction pathways in compression-induced responses, inhibition experiments were performed using *Piezo1* and *YAP/TAZ* inhibitors (Figure 3I). In the *Piezo1* inhibitor-treated group, compression-induced *Piezo1* expression was markedly reduced to a level comparable to that of the non-compressed control (Figure 3J-K). Correspondingly, the expression of the ECM remodeling-associated markers *EGFR*, *MMP2*, and *MMP9* was also significantly decreased. A similar trend was observed following *YAP/TAZ* inhibitor treatment, where compression-induced upregulation of *YAP*, *TAZ*, *EGFR*, *MMP2*, and *MMP9* was substantially attenuated (Figure S3). Collectively, these findings support the involvement of Piezo1- and YAP/TAZ-associated mechanotransduction pathways in compression-induced oncogenic signaling and ECM remodeling.

**Figure 4:**
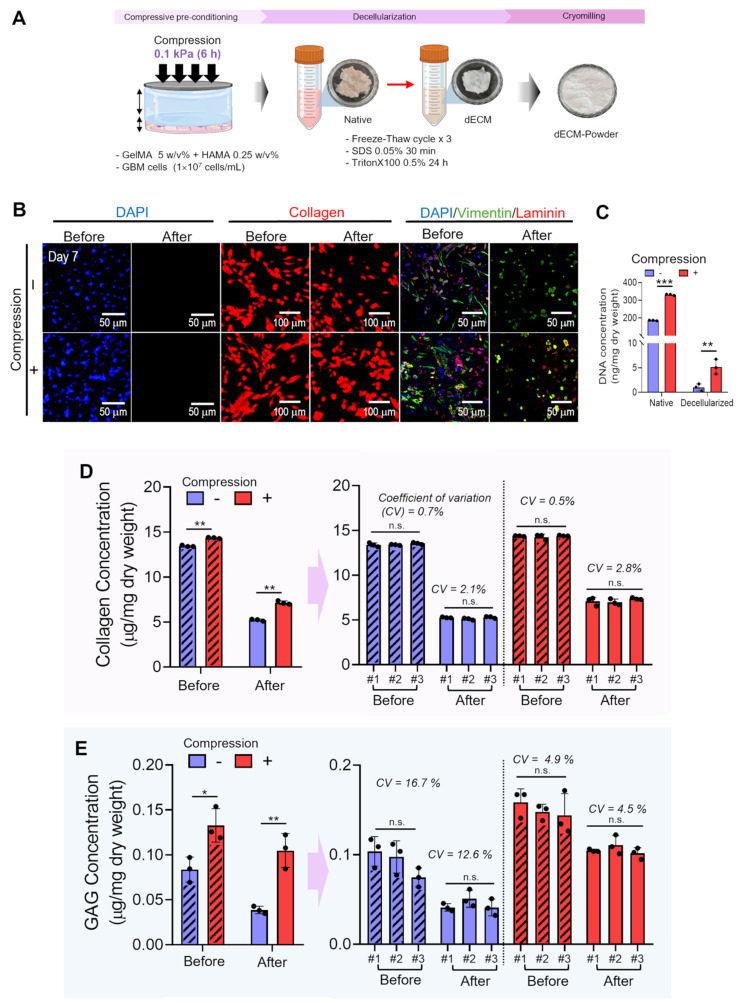
** Fabrication and biochemical validation of compression-conditioned GBM-derived dECM powder. (A)** Workflow for producing compression-conditioned dECM (C-dECM) powder, including mechanical pre-conditioning of GBM-laden constructs, decellularization, and subsequent cryomilling to obtain ECM powder. **(B)** Immunofluorescence images of DAPI (blue), collagen (red), vimentin (green), and laminin (red) before and after decellularization. **(C)** Quantitative DNA analysis (n = 3) demonstrating substantial reduction of residual DNA to levels meeting established decellularization criteria. **(D,E)** Quantification of collagen and sulfated glycosaminoglycan (GAG) contents (n = 3), demonstrating retention of ECM components and batch-to-batch reproducibility of dECM recovery based on three independent repetitions. Student's t-test was used for two-group comparisons, and one-way ANOVA followed by Tukey's HSD post-hoc test was used for multiple comparisons.

Figure 4A illustrates the decellularization of compressed GBM-laden hydrogel. GBM cells are known to predominantly deposit a tumor-specific ECM enriched in fibrillar collagens (mainly collagen I and III), basement membrane–associated proteins (collagen IV, laminin), and sulfated glycosaminoglycans (GAGs), all of which are involved in tumor mechanotransduction and growth factor sequestration 61, 62. Following ECM deposition, a combined freeze–thaw and mild detergent protocol (0.05% SDS and 0.5% Triton X-100) was applied to remove cellular components while preserving ECM constituents. DAPI staining (Figure 4B) revealed abundant nuclei in native cell–ECM constructs (Before decellularization), regardless of compressive preconditioning (- and +), whereas nuclear signals were nearly absent after decellularization, indicating effective cell removal. DNA quantification further showed a pronounced reduction in DNA content in both non-compressed dECM (-) and compressed dECM (+), reaching levels consistent with established decellularization criteria (<50 ng DNA mg⁻¹ dry ECM) (Figure 4C) 63.

Immunofluorescence analysis revealed that the expression of major tumor ECM components, including collagen and laminin, and cytoskeleton protein, vimentin, was reduced following decellularization, as expected due to detergent exposure, yet remained clearly detectable, demonstrating preservation of dECM components (Figure 4B). The extent of ECM component retention was comparable between compressed and non-compressed groups. In addition, collagen and GAG contents were quantified from three independently prepared GBM-derived dECM batches, each generated through separate cycles of cell culture, compression, and decellularization and measured in triplicate, to assess the reproducibility of GBM-dECM production (Figure 4D-E). As shown in Figure 4D, collagen content exhibited minimal batch-to-batch variation, with a coefficient of variation (CV) below 3%, indicating highly consistent ECM retention regardless of compression. In contrast, GAG levels showed moderately greater variability (CV 5–17%, Figure 4E), but the overall variation remained within an acceptable range. Taken together, the marked reduction in nuclear DNA together with the retained presence of collagen, vimentin, laminin, and GAGs confirms that the cell-derived ECM was successfully decellularized while possibly maintaining key biochemical cues characteristic of the GBM tumor microenvironment. These results also show that our platform produces substantially more consistent and reproducible dECM quantities than conventional tissue-derived dECM.

### A cytocompatible bioprinting window for 3D GBM constructs

**Figure 5:**
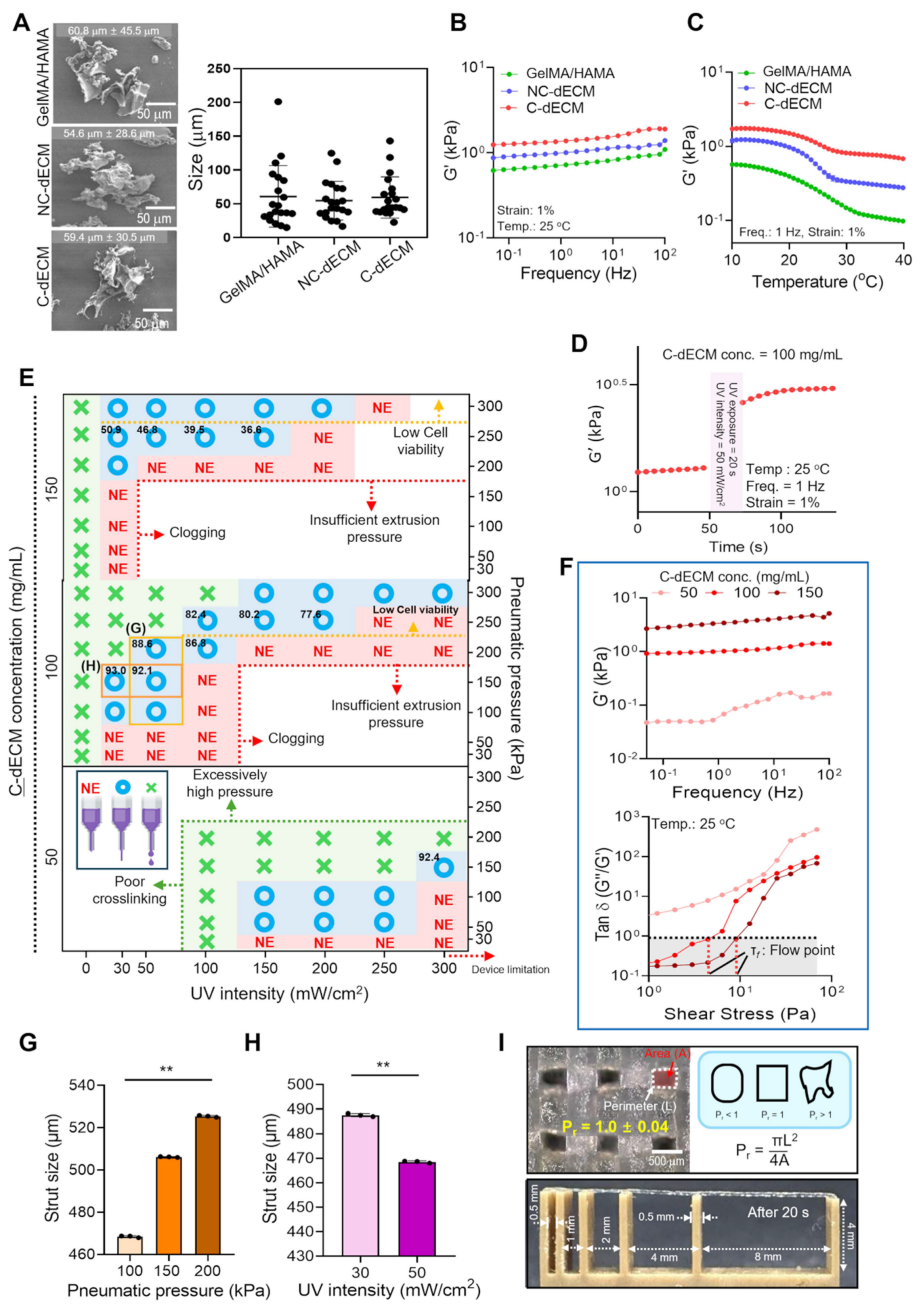
** A cytocompatible bioprinting window for GelMA/dECM composite bioinks enabling stable 3D mesh fabrication. (A)** SEM images of cryomilled powders (control, NC-dECM, and C-dECM), showing irregular flake-like microstructures and corresponding size distribution (n = 20). **(B,C)** Storage modulus (G′) for frequency and temperature sweeps, showing concentration-dependent mechanical reinforcement with increasing dECM content while preserving the thermoresponsive gel–sol transition of GelMA hydrogels. **(D)** Time-sweep rheological analysis of C-dECM/GelMA hydrogel (100 mg mL⁻¹ C-dECM) under UV exposure (20 s, 50 mW cm⁻²) after UV exposure. **(E)** Printability maps as a function of C-dECM concentration, pneumatic pressure, and UV intensity, categorizing non-extrusion (NE), unstable (×), and stable (●) regimes with corresponding post-printing cell viability. **(F)** G′ and tan δ of composite bioinks containing varying C-dECM concentrations (50–150 mg mL⁻¹), across the printable concentration range. **(G,H)** Strut diameters (n = 3) under selected stable conditions (C-dECM 100 mg mL⁻¹; pneumatic pressure 100–200 kPa; UV intensity 30 and 50 mW cm⁻²), with corresponding cell viability. **(I)** Optical images of printed mesh structures fabricated under selected conditions, showing high print fidelity (printability, P_r_ ≈ 1) and successful bridging tests. Student's t-test was used for two-group comparisons, and one-way ANOVA followed by Tukey's HSD post-hoc test was used for multiple comparisons.

To enable their use as bioink components, GBM-derived dECM and hydrogel materials were lyophilized and processed into powders. For stable extrusion-based bioprinting, these powders were subsequently into a GelMA matrix to formulate a composite bioink. Specifically, cryomilled dECM/GelMA/HAMA powder was blended with 1 w/v% GelMA hydrogel at concentrations of 50, 100, and 150 mg mL⁻¹. The resulting composite bioinks were then loaded into the bioprinter and extruded under the printing conditions shown in Figure S4A. As a control, pure GelMA/HAMA was cryomilled and subsequently incorporated into a GelMA (1 w/v%) matrix hydrogel. SEM imaging and flake-size analysis showed that both the control and microscale dECM powders (NC-dECM: non-compression and C-dECM: compression) had irregular, flake-like microstructures (Figure 5A). After incorporation of the microscale dECM powder into the GelMA hydrogel, optical and SEM images revealed no substantial morphological differences among the control, NC-CB, and C-CB groups (Figure S4B). Rheological data showed that incorporation of dECM-based powders increased the storage modulus (G′) compared with the control (GelMA/HAMA powder), indicating that GBM-derived dECM components may act as a mechanically reinforcing phase (Figure 5B). This effect is likely related to the presence of fibrous ECM proteins (*e.g.*, collagens) and GAGs, which introduce additional load-bearing elements and restrict polymer chain mobility within the GelMA network. Notably, GelMA containing compressed dECM powder (C-dECM) exhibited a substantially higher G′ than the NC-dECM powder.

In addition, all hydrogels displayed the characteristic thermoresponsive gel–sol transition behavior of GelMA during the temperature sweep (Figure 5C). Time-sweep analysis under UV irradiation (50 mW cm⁻² for 20 s) further confirmed efficient photocrosslinking of the C-dECM–laden hydrogel, as evidenced by the rapid increase and stabilization of the storage modulus (Figure 5D). In addition, the stress–strain curves showed that both NC-CB and C-CB constructs generated higher stress values than the control at the same applied strain (Figure S4C). Similarly, the compressive modulus was significantly higher in both dECM-containing groups than in the control (Figure S4D), while all measured values remained within the reported stiffness range of native GBM tissue. These results are consistent with the rheological analysis, indicating that incorporation of dECM improved the mechanical properties of the constructs. Although the C-CB group exhibited a higher storage modulus than the NC-CB group in the rheological analysis, no significant difference in compressive modulus was observed. This may be because the bulk compressive stiffness of the crosslinked GelMA/HAMA hydrogel was mainly influenced by the hydrogel network, thereby minimizing the contribution of the differences between the incorporated dECM powders.

To construct a physiologically relevant *in vitro* GBM model, it is essential to satisfy two competing requirements including: (i) sufficient macroscopic volume to enable long-term culture and experimental manipulation, and (ii) adequate short diffusion distances to maintain homogeneous cell viability throughout the construct 64. In bulk constructs, steep diffusion gradients can arise, leading to hypoxia, nutrient deprivation, and progressive necrosis in the core region, which is particularly problematic for metabolically active and rapidly proliferating GBM cells 65. Unlike conventional bulk constructs, 3D-printed mesh architectures consist of microscale struts and interconnected pores that shorten diffusion distances and facilitate molecular transport of oxygen, nutrients, and metabolic waste. This architecture also supports a more uniform distribution of cells, mechanical cues, and ECM organization. These structural characteristics are well suited for reproducing key features of the GBM microenvironment and tumor–microenvironment interactions 66. Based on these considerations, a strut diameter of 400–500 µm was selected because it maintained print fidelity and structural stability without compromising molecular diffusion or cell viability 67-70.

Based on this design, we mapped the printability window of composite bioinks containing C-dECM powder at varying concentrations (50, 100, and 150 mg mL⁻¹), as a function of UV intensity and pneumatic pressure (Figure 5E). The printing outcomes were categorized into three regimes: non-extrusion (NE), unstable extrusion (×), and stable filament formation (●), while the black numerical values in each condition indicate the post-printing cell viability. At low pressures and/or high UV intensities, the bioink failed to extrude due to premature gelation or insufficient driving force, resulting in widespread NE regions. Conversely, at excessively high pressures and low crosslinking intensities, unstable or discontinuous filaments were frequently observed, reflecting insufficient shape fixation during deposition. At the highest powder concentration (150 mg mL⁻¹), stable printing was still achieved; however, cell viability was markedly reduced (below 60%), likely due to the increased G’ and flow point (τ_f_) shown in Figure 5F. In contrast, the 50 mg mL⁻¹ formulation, while easier to extrude, exhibited a relatively limited regime of mechanically stable printing, indicating insufficient structural integrity after deposition. Among the tested conditions, the 100 mg mL⁻¹ dECM-powder formulation showed the most balanced printability window, with stable filament formation across a broad range of conditions with consistently high cell viability.

Within this selected composition (100 mg mL⁻¹), two representative stable-printing conditions, designated as (G) and (H) in Figure 5E, were selected for detailed further evaluation of cell-laden strut geometry (Figure 5G-H). These two conditions represented different combinations of UV intensity and pneumatic pressure within the stable printing window. Both produced continuous and well-defined filaments, although clear differences in strut dimensions were observed. Under condition (G), increasing the pneumatic pressures from 100 to 200 kPa at a fixed UV intensity of 50 mW cm^-^² increased the strut diameter from 468 μm to 525 μm (Figure 5G). In contrast, condition (H), which was printed at 150 kPa with a higher UV intensity, generated thinner struts with an average diameter of approximately 470 μm. Cell viability remained high under both conditions (89–95%), indicating that the printing parameters used in this study did not cause measurable cytotoxicity. Based on these results, the final printing condition was set using a 100 mg mL⁻¹ dECM-based bioink at 100 kPa and 50 mW cm⁻², which yielded a particularly high cell viability of 95% and stable GBM-laden strut size (470 μm).

Mesh structures were fabricated under the selected printing condition, and their print fidelity and structural stability were evaluated using printability (P_r_) analysis and bridging tests (Figure 5I). The printed grids exhibited P_r_ values very close to 1, indicating that the deposited filaments formed nearly perfect square pores without over-spreading (P_r_ < 1) or filament thinning and discontinuity (P_r_ > 1). In the bridge test, filaments spanning unsupported gaps maintained their shape with little sagging or collapse, demonstrating sufficient mechanical stability immediately after printing. These results suggest that the selected bioink and printing parameters enable reliable fabrication of well-defined multilayered mesh structures with controlled pore networks and reproducible microarchitecture 66, 71.

### Characterization and evaluation of a bioprinted composite bioink for *in vitro* GBM modeling

**Figure 6:**
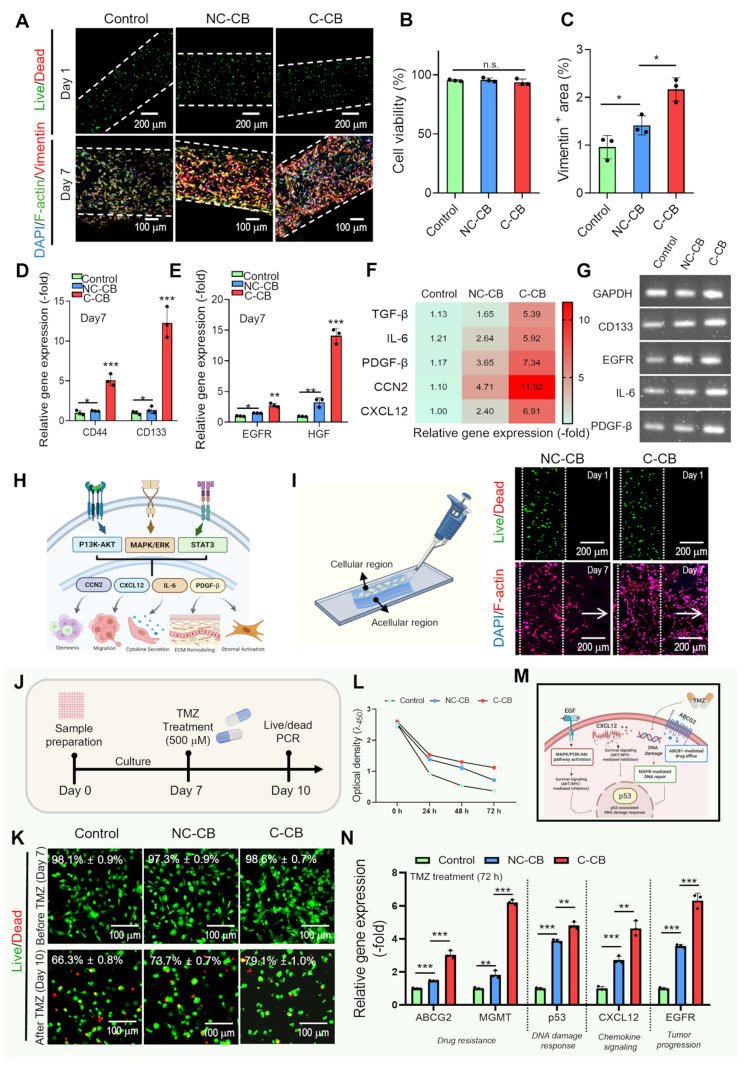
** Biological performance of bioprinted GelMA/dECM composite constructs for *in vitro* GBM modeling. (A)** Live/Dead images at day 1 demonstrating uniformly high cell viability across printed constructs and DAPI/F-actin (green)/vimentin (red) staining at day 7 showing enhanced cytoskeletal organization and increased vimentin expression in dECM-containing groups, most prominently in C-dECM (C-CB) constructs. **(B)** Quantified cell viability (n = 3) after printing (>93–95% survival). **(C)** Quantification of vimentin⁺ area (n = 3). **(D,E)** Gene expression analysis of glioma stem-like markers (*CD44*, *CD133*) and oncogenic growth factor signaling components (*EGFR*, *HGF*) in control, NC-CB, and C-CB constructs (n = 3). **(F)** Heat map of GBM-associated genes (*e.g.*, *TGF-β, IL-6, PDGF-β, CCN2, CXCL12*) (n = 3). **(G)** Representative RT-qPCR electrophoresis. **(H)** Schematic of growth factor–mediated *PI3K–AKT/MAPK/STAT3* pathways associated with stemness, invasion, and stromal remodeling. **(I)** Schematic and representative images of the 3D invasion assay consisting of adjacent cellular and acellular regions. Live/Dead staining on day 1 and DAPI (blue)/F-actin (red) staining on day 7. White arrows indicating the direction of cell invasion. **(J)** Experimental timeline of the temozolomide (TMZ) response assay. **(K)** Live/Dead images before TMZ treatment on day 7 and after 72 h of TMZ treatment on day 10. **(L)** Time-dependent changes in cell proliferation of the control, NC-CB, and C-CB constructs at 0, 24, 48, and 72 h after TMZ treatment (n = 3), measured by CCK assay. **(M)** Schematic illustrating TMZ-induced DNA damage, *p53*-associated stress responses, and major drug-resistance mechanisms. **(N)** Relative expression of the drug resistance-associated genes (*ABCG2* and *MGMT*), the DNA damage response-associated gene (*p53*), the chemokine signaling-associated gene *CXCL12*, and the oncogenic signaling-associated gene *EGFR* after 72 h of TMZ treatment (n = 3). One-way ANOVA followed by Tukey's HSD post-hoc test was used for multiple comparisons.

Using the selected printing parameters and microscale mesh architecture described above, we evaluated the biological performance of three different constructs: a control GelMA (1 w/v%) hydrogel containing GelMA/HAMA powder (100 mg mL^-1^) without dECM, a composite GelMA bioink with NC-dECM powder (100 mg mL^-1^) (NC-CB), and a composite GelMA bioink with C-dECM powder (100 mg mL^-1^) (C-CB). Live/Dead staining at day 1 revealed uniformly high survival in all groups, with quantitative viabilities exceeding ~93–96%, confirming that the selected bioprinting condition was safe (Figure 6A-B).

Despite comparable viability, pronounced differences were observed in cytoskeletal organization. DAPI/F-actin (green)/vimentin (red) staining at day 7 revealed weak and sparse vimentin signal in the control construct, whereas both dECM-containing groups showed markedly enhanced vimentin-positive regions, with the C-CB construct exhibiting the strongest and most extensive expression (Figure 6A,C). Because vimentin is a representative marker of mesenchymal-like, highly invasive GBM phenotypes and is closely associated with increased motility, cytoskeletal plasticity, and poor prognosis 72, this result suggests that the C-CB construct promotes a more clinically relevant malignant phenotype rather than simply supporting cell survival.

These phenotypic differences are consistent with the transcriptional profiles (Figure 6D-F). Compared to the control, both biocomposite groups—most prominently the C-CB constructs—showed coordinated upregulation of key GBM-associated genes, including *CD44*, *CD133*, *EGFR*, and *HGF*. Detailly, *CD44*, a principal mediator of cell–matrix interactions and invasion, was elevated in parallel with vimentin, supporting enhanced mesenchymal-like behavior, and *CD133*, a representative glioma stem-like cell marker, was also increased, indicating that the tumor-derived ECM environment may support stem-like, highly tumorigenic subpopulations (Figure 6D) 62. In addition, the upregulation of *EGFR* and its cooperating ligand *HGF* can suggest activation of oncogenic growth factor signaling pathways that drive proliferation, invasion, and therapeutic resistance in native GBM (Figure 6E) 73.

As summarized in the schematic in Figure 6F, tumor-derived signals such as *TGF-β*, *EGFR* ligands, and *HGF* are linked to downstream pathways including *PI3K*–*AKT*, *MAPK*/*ERK*, and *STAT3*. These signaling networks are associated with increased expression of *CCN2*, *CXCL12*, *IL-6*, and *PDGF-β*, which are known to support stem-like phenotypes, enhancing migratory and invasive capacity, amplifying cytokine secretion, promoting ECM remodeling, and driving stromal activation within the reconstructed TME. In line with this scheme, Figure 6G and Figure S5 showed coordinated upregulation of *TGF-β*, *IL-6*, *PDGF-β*, *CCN2*, and *CXCL2* in the C-CB construct, consistent with the signaling pathways illustrated in Figure 6F 74-76. These results indicate that the C-CB construct reproduced tumor-associated signaling more strongly than the control and NC-CB groups and was accompanied by molecular changes linked to GBM aggressiveness *via* integrin-mediated mechanotransduction and autocrine cytokine signaling. Consistent with the RT-qPCR results, the electrophoresis image of Figure 6H shows markedly stronger bands for *CD133, EGFR, IL-6,* and* PDGF-β* in the C-CB construct compared to the control and NC-CB, supporting the increased transcript levels observed by gene expression analysis.

To further evaluate the functional characteristics of the developed GBM models, invasion and drug resistance assays were performed. First, a 3D invasion assay was conducted using constructs composed of adjacent cellular and acellular regions (Figure 6I). Live/Dead staining on day 1 confirmed high cell viability within the cellular region after bioprinting. By day 7, DAPI/F-actin staining revealed migration of GBM cells across the original interface into the acellular region in both dECM-containing constructs. The C-CB group exhibited more extensive invasion than the NC-CB group, suggesting enhanced invasive behavior under the compressed dECM condition.

A similar trend was observed in the drug resistance assay. The constructs were cultured for 7 days, treated with TMZ for 72 h, and subsequently evaluated for metabolic activity, cell viability, and treatment response-associated gene expression (Figure 6J). Live/Dead staining showed high cell viability before TMZ treatment, followed by an increase in dead cells after drug exposure (Figure 6K). However, both dECM-containing groups maintained higher cell viability than the control, with the C-CB group exhibiting the highest viability after TMZ treatment. Similarly, comparison of cell proliferation over time demonstrated that the C-CB group exhibited the smallest reduction in cell proliferation among all experimental groups following TMZ treatment (Figure 6L).

The schematic in Figure 6M summarizes the major cellular responses associated with TMZ treatment 77-81. Following TMZ exposure, DNA damage induces *p53*-mediated stress signaling, while resistance mechanisms such as *ABCG2*-mediated drug efflux and *MGMT*-mediated DNA repair can reduce the intracellular cytotoxic effects of TMZ. In parallel, tumor-associated signaling pathways remain active, as reflected by increased *CXCL12*- and *EGFR*-associated signaling, which may contribute to tumor progression and cell survival during TMZ treatment. In line with this mechanism, RT-qPCR analysis showed that the C-CB construct exhibited increased expression of the drug resistance-associated genes (*ABCG2 and MGMT*) together with the DNA damage response marker *p53*, as well as the chemokine signaling-associated gene *CXCL12* and the tumor progression-associated gene *EGFR*, followed by the NC-CB and control groups (Figure 6N). These results suggest activation of both TMZ-induced stress responses and resistance-associated signaling in the C-CB construct.

Collectively, these functional results are consistent with the enhanced malignant phenotype observed in the C-CB construct, including increased invasion, elevated tumor-associated gene expression, and reduced sensitivity to TMZ treatment. These findings suggest that compression-conditioned dECM promotes a more aggressive and drug-resistant GBM phenotype.

### Multicellular GBM-TME modeling through fibroblast or endothelial co-culture

Although fibroblasts are not a major stromal population in the normal brain, they were included in the co-culture model to evaluate whether GBM-derived biochemical and mechanical cues could induce a CAF-like activation program 82, 83. This strategy enables a mechanistic validation of GBM-driven stromal reprogramming, particularly in terms of ECM remodeling and pro-tumorigenic niche formation, rather than claiming a direct cellular origin of GBM-associated CAF-like cells.

**Figure 7:**
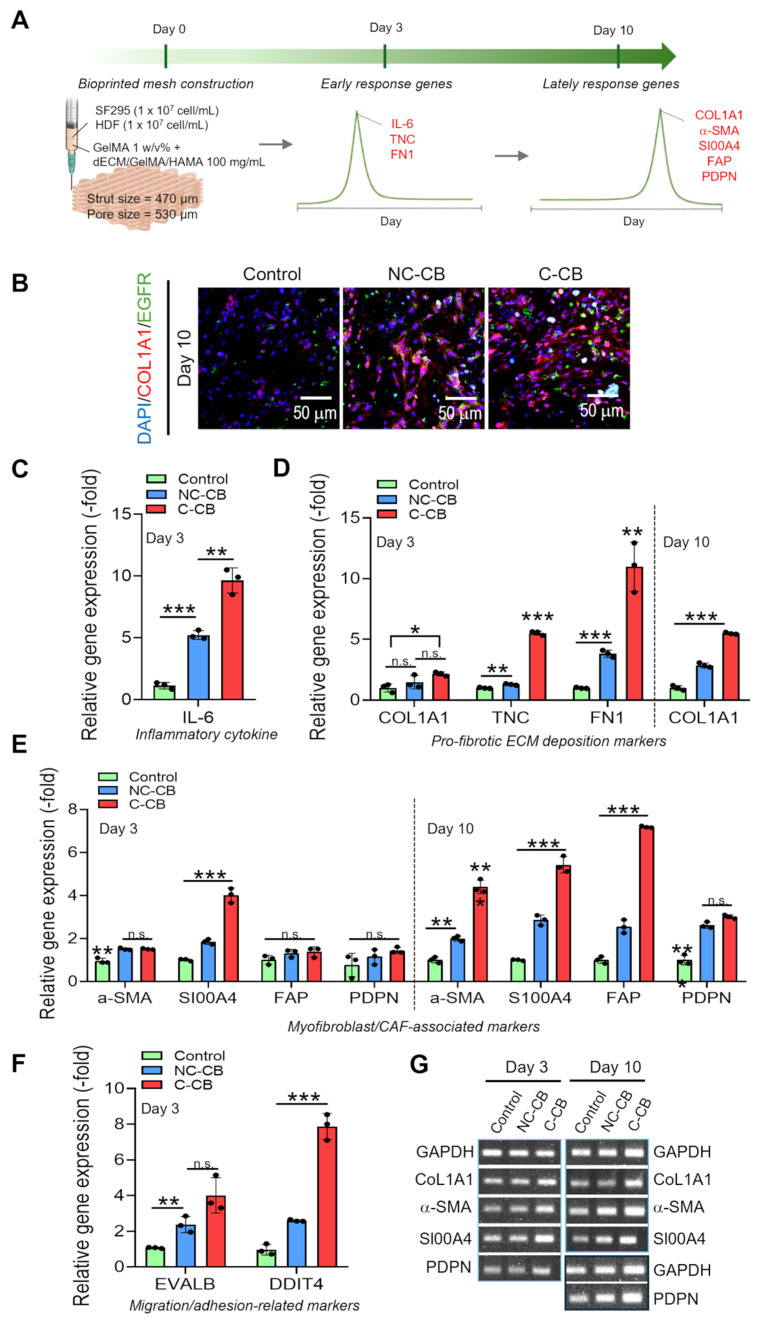
** Fibroblast co-culture revealing CAF-like stromal activation in compression-conditioned GBM TME constructs. (A)** Schematic illustration of multicellular GBM–TME modeling *via* co-culture of GBM cells with human dermal fibroblasts (HDFs) in bioprinted mesh constructs. **(B)** DAPI (blue)/COL1A1 (green)/EGFR (red) immunofluorescence images of GBM–fibroblast co-culture constructs at day 10. **(C-F)** RT-qPCR analysis of inflammatory cytokines (IL-6), pro-fibrotic ECM markers (COL1A1, FN1, TNC), and CAF-associated genes (*α-SMA, S100A4, FAP, PDPN*), demonstrating significant upregulation in C-CB constructs compared to control and NC-CB groups (n = 3). **(G)** Agarose gel electrophoresis. One-way ANOVA followed by Tukey's HSD post-hoc test was used for multiple comparisons.

To evaluate whether the C-CB construct enhances stromal activation and tumor–stroma interactions, GBM cells (1×10^7^ cells/mL) were co-cultured with human dermal fibroblasts (HDFs, 1×10^7^ cells/mL). Figure 7A depicts the workflow for multicellular GBM–TME modeling *via* fibroblast co-culture. A 3D bioprinted mesh construct was co-cultured with fibroblasts, leading to sequential activation of inflammatory/ECM markers (*IL-6, TNC, FN1*) followed by CAF-associated genes (*COL1A1, α-SMA, S100A4, FAP, PDPN*), indicating progressive stromal activation.

Immunofluorescence staining for COL1A1 at day 10 showed that both dECM-containing groups exhibited increased COL1A1 expression compared with the control (Figure 7B). Notably, the C-CB constructs showed the highest COL1A1 expression among the three groups, indicating enhanced stromal activation. Consistent with the immunofluorescence results, RT-qPCR analysis (Figure 7C-F) and electrophoresis result (Figure 7G) showed significant upregulation of TME-associated genes in the C-CB group, including the inflammatory cytokine *IL-6*, pro-fibrotic ECM deposition markers (*COL1A1*, *FN1*, *TNC*), myofibroblast/CAF-associated markers (*α-SMA*, *S100A4*, *FAP*, *PDPN*), and migration- and adhesion-related genes (*EVALB*, *DDIT4*). These transcriptional changes are likely attributable to the increased ECM ligand density and compact fibrous architecture of the C-CB construct, which promote integrin clustering and subsequent activation of mechanotransductive pathways (*e.g.*, *FAK/YAP/TAZ*). This enhanced mechanosignaling cascade may further stimulate autocrine and paracrine cytokine secretion, thereby reinforcing stromal activation and CAF-like phenotypic remodeling of the microenvironment 84, 85. Collectively, these results suggest that the C-CB construct more faithfully recapitulates key hallmarks of the pathological GBM tumor microenvironment—including CAF-like activation, ECM remodeling, and inflammatory signaling—supporting its potential as a biomimetic and physiologically relevant *in vitro* TME platform for modeling GBM progression and therapeutic responses.

**Figure 8:**
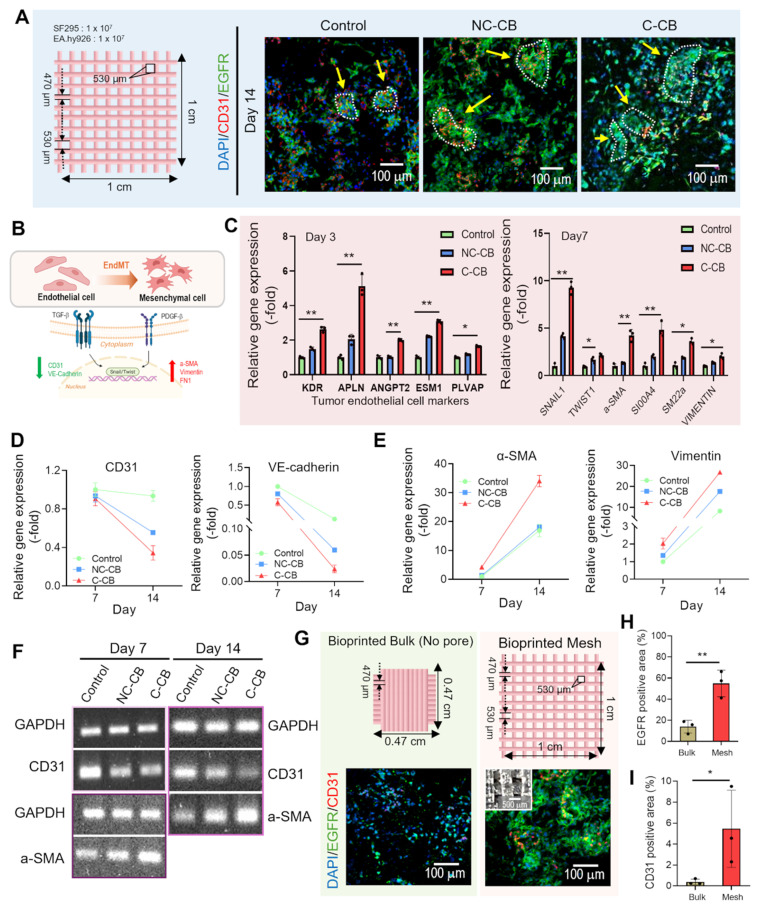
** Compression-conditioned dECM promoting endothelial plasticity and architecture-dependent tumor–vascular interactions in 3D GBM constructs. (A)** Experimental design and representative immunofluorescence images of GBM (SF295) and endothelial cell (EA.hy926) co-culture constructs (Day 14). DAPI (blue)/CD31 (red)/EGFR (green) staining showing enhanced EGFR expression and more aggregation of the GBM cells in C-CB groups compared with control and NC-CB. Yellow arrows indicate aggregated tumor regions. **(B)** Schematic illustration of endothelial-to-mesenchymal transition (EndMT), characterized by decreased endothelial markers (*CD31, VE-cadherin*) and increased mesenchymal markers (*α-SMA, Vimentin, FN1*) *via* Snail/Twist-mediated transcriptional reprogramming. **(C)** Tumor-associated endothelial activation and mesenchymal gene expression (n = 3). **(D,E)** Quantitative analysis of endothelial and mesenchymal gene expression demonstrating downregulation of *CD31/VE-cadherin* and upregulation of mesenchymal/TME-associated markers (*α-SMA and vimentin*) in C-CB constructs over time (n = 3). **(F)** RT-qPCR electrophoresis. **(G)** Schematic of bioprinted bulk and mesh architectures for GBM–EA co-culture constructs and DAPI/EGFR/CD31 staining images of bulk and mesh constructs with an optical image of mesh structures, showing more extensive cellular networks and angiogenic interactions in mesh geometry. **(H,I)** Quantification of EGFR⁺ and CD31⁺ areas (n = 3), demonstrating significantly enhanced tumor activity and endothelial organization in mesh constructs compared with bulk hydrogels. Student's t-test was used for two-group comparisons, and one-way ANOVA followed by Tukey's HSD post-hoc test was used for multiple comparisons.

In addition, we hypothesized that the GBM secretome, particularly under mechanically stressed conditions, is sufficient to induce an endothelial-to-mesenchymal transition (EndMT), thereby converting endothelial cells into CAF-like stromal cells that actively remodel the tumor ECM. This process is particularly relevant in GBM, where the perivascular niche represents a dominant structural and signaling hub for tumor invasion and mechanoadaptation 86. Therefore, incorporating EndMT-driven CAF-like stromal conversion is essential to recapitulate the dynamic and self-reinforcing nature of the GBM tumor microenvironment *in vitro*. To investigate whether the C-CB construct enhances vascular–tumor interactions within the engineered microenvironment, GBM cells (1×10^7^ cells mL^-1^) were co-cultured with endothelial cells (EA.hy926, 1×10^7^ cells mL^-1^) followed by DAPI/CD31 (red)/EGFR (green) immunofluorescence analysis at 14 days (Figure 8A). Notably, both NC-CB and C-CB constructs displayed reduced CD31-positive endothelial signals together with increased α-SMA-positive regions, indicating endothelial phenotypic changes toward a mesenchymal-like state (Figure 8A and Figure S6A). In contrast, EGFR expressions in GBM cells was significantly higher than in the control group and was accompanied by pronounced cellular aggregation (yellow arrows). CD31 staining was reduced in the endothelial cell population, whereas EGFR expression increased in adjacent GBM cells. Together, these changes indicate endothelial phenotypic alteration accompanied by enhanced tumor cell activity in the compressed dECM microenvironment. These findings are also consistent with stronger tumor–stromal interactions in the engineered TME.

The proposed mechanism is summarized in Figure 8B, where endothelial cells undergo endothelial-to-mesenchymal transition (EndMT), progressively acquiring mesenchymal/stromal cell–like phenotypes characterized by reduced endothelial markers (*CD31, VE-cadherin*) and increased mesenchymal markers (*α-SMA, FN1, Vimentin, SNAI1/2*). Consistent with this model, GBM/EA co-culture for 3 and 7 days increased the expression of tumor endothelial cell (TEC)–associated and EndMT-related genes in C-CB construct compared with the control and NC-CB constructs (Figure 8C). Genes involved in angiogenic signaling and vascular remodeling were also upregulated. The quantitative heat map further showed a condition-dependent increase (≈1.4–3.5-fold) in TEC/angiogenic-related gene signatures in endothelial cells cultured with GBM cells. These changes were more consistent with partial endothelial reprogramming toward a tumor-conditioned, pro-angiogenic (TEC-like) phenotype than with complete conversion into TECs (Figure S6B).

RT-qPCR and electrophoresis analyses performed on days 7 and 14 showed reduced expression of endothelial markers together with increased expression of mesenchymal and TME-associated markers (Figure 8D–F). These transcriptional changes are consistent with partial phenotypic reprogramming of endothelial cells exposed to the compressed dECM, accompanied by the acquisition of mesenchymal stromal-like features. The compressed dECM microenvironment likely contributed to this transition by promoting EndMT-associated changes and modifying paracrine signaling, which are closely linked to vascular remodeling during GBM progression.

To compare the effects of bulk *vs.* mesh TME architectures, GBM/EA co-culture constructs were fabricated using C-CB bioink in either bioprinted bulk or mesh hydrogels, shown in Figure 8G. After 7 days of co-culture, the two structures differed in the way tumor and endothelial cells were organized. Mesh constructs showed significantly greater EGFR⁺ and CD31⁺ positive areas than bulk hydrogels (Figure 8G-I). In the immunofluorescence images, cells in the mesh constructs formed more extensive, spatially interconnected, and densely aggregated cellular networks within the mesh architecture, whereas bulk constructs exhibited comparatively confined and fragmented cellular distributions. Quantitative analysis confirmed that both tumor-associated EGFR expression and endothelial CD31 coverage were substantially elevated in the mesh group. To examine whether these structural differences were accompanied by differences in transport behavior, rhodamine B was incorporated into the C-CB bioink, and its release from bulk and mesh constructs into the surrounding solution was monitored for 30 min (Figure S7A-B). The mesh constructs exhibited a more rapid increase in the absorbance of the surrounding solution than the bulk constructs, indicating faster rhodamine B diffusion. In addition, *HIF-1α* gene expression was significantly lower in the mesh constructs than in the bulk constructs (Figure S7C). These results suggest that the mesh structure clearly improves mass transport, reduces diffusion-limited hypoxia, and increases effective surface area for heterotypic cell–cell interactions.

The present results demonstrate that mechanically regulated cell-derived dECM can provide a controllable and reproducible strategy for generating GBM-associated extracellular matrix cues, thereby improving the physiological relevance of hydrogel-based GBM models. Nevertheless, several limitations of the present study should be acknowledged. Although the proposed preparation strategy substantially improved the controllability and reproducibility of GBM-derived dECM compared with conventional tissue-derived dECM, batch-to-batch variations associated with cell culture conditions, cell passage, lot-to-lot variations of biomaterials and culture reagents (e.g., GelMA, HAMA, and FBS), ECM deposition, and decellularization efficiency cannot be completely eliminated. In addition, the present platform employed static compressive stimulation to regulate GBM-derived ECM production, whereas the native GBM microenvironment is exposed to dynamic and heterogeneous mechanical cues. Furthermore, the current study was performed using established GBM, endothelial, and fibroblast cell lines, which cannot fully recapitulate the biological heterogeneity of patient-derived tumors and their surrounding stromal microenvironment. In addition, although endothelial phenotypic changes and angiogenesis-related responses were evaluated via gene expression analysis and immunostaining, functional microvascular network formation was not investigated in the present study. Future studies incorporating patient-derived cells, vascularized models, dynamic mechanical stimulation, and immune cells will extend the current platform to develop more biologically representative GBM models, thereby supporting personalized therapeutic evaluations and GBM-on-chip applications.

## Conclusion

In this study, we developed a GBM-derived decellularized extracellular matrix (dECM) bioink that was designed to better preserve tumor-relevant biochemical cues while reducing the batch-to-batch variability and reproducibility issues often associated with conventional tissue-derived dECM. By incorporating dECM derived from 3D GBM cultures exposed to a physiologically relevant compressive stimulus (0.1 kPa), the bioink was enriched with tumor-associated biochemical cues and promoted a more aggressiveness GBM phenotype, including increased invasion-related gene expression, and ECM remodeling compared with hydrogel-only controls. This strategy overcomes the limited controllability and reproducibility of conventional tissue-derived dECM while integrating the biochemical advantages of cell-derived dECM with the tunability and fabrication versatility of hydrogel-based bioinks. Micromesh-bioprinted constructs maintained their printed architecture during culture and supported stromal co-culture with endothelial cells or fibroblasts. The resulting models reproduced stromal activation, paracrine communication, and matrix-dependent mechanotransduction observed in the GBM tumor microenvironment. Although further validation using patient-derived GBM cells, multiple therapeutic agents, and chip-integrated culture systems is required, this platform establishes a mechanically conditioned and physiologically relevant *in vitro* bioink platform, serving as a promising foundation for future GBM-on-chip applications and providing an initial platform for mechanobiology studies and therapeutic evaluation.

## Supplementary Material

Supplementary figures and tables, including Figure S8, which provides the raw gel electrophoresis images corresponding to each figure.

## Data Availability

The data that support the findings of this study are available from the corresponding author upon reasonable request.

## Abbreviations

GBM: Glioblastoma
dECM: Decellularized extracellular matrix
GelMA: Gelatin methacryloyl
HAMA: Hyaluronic acid methacrylate
TME: Tumor microenvironment
DAPI: 4′,6-diamidino-2-phenylindole
PIEZO1: Piezo-type mechanosensitive ion channel component
YAP: Yes-associated protein
TAZ: Transcriptional co-activator with PDZ-binding motif
GDF15: Growth differentiation factor 15
MMP2: Matrix metalloproteinase 2
MMP9: Matrix metalloproteinase 9
RUNX1: Runt-related transcription factor 1
F-actin: Filamentous actin
Ki67: Marker of proliferation Ki-67
HIF-1α: Hypoxia-inducible factor 1 alpha
COL4A1: Collagen type IV alpha 1
C-dECM: Compression-conditioned decellularized extracellular matrix
GAG: Glycosaminoglycan
SEM: Scanning electron microscopy
NC-dECM: Non-compressed decellularized extracellular matrix
NE: Non-extrusion
Pr: Printability
NC-CB: Non-compressed dECM-containing composite bioink
C-CB: Compression-conditioned dECM-containing composite bioink
EGFR: Epidermal growth factor receptor
HGF: Hepatocyte growth factor
PI3K: Phosphoinositide 3-kinase
AKT: Protein kinase B
MAPK: Mitogen-activated protein kinase
STAT3: Signal transducer and activator of transcription 3
TGF-β: Transforming growth factor beta
IL-6: Interleukin 6
PDGF-β: Platelet-derived growth factor beta
CCN2: Cellular communication network factor 2
CXCL12: C-X-C motif chemokine ligand 12
CAF: Cancer-associated fibroblast
HDFs: Human dermal fibroblasts
COL1A1: Collagen type I alpha 1
FN1: Fibronectin 1
TNC: Tenascin C
α-SMA: Alpha-smooth muscle actin
S100A4: S100 calcium-binding protein A4
FAP: Fibroblast activation protein
PDPN: Podoplanin
CD31: Cluster of differentiation 31
EA.hy926: Human endothelial cell line EA.hy926
EndMT: Endothelial-to-mesenchymal transition
VE-cadherin: Vascular endothelial cadherin
TMZ: Temozolomide
ABCG: ATP-Binding Cassette sub-family G member 2
MGMT: ATP-Binding Cassette sub-family G member 2
P53: TP53
