# Supplementary material for: A mechanobiology-driven cell-derived ECM bioink for engineering 3D glioblastoma tumor microenvironment models
Source: Theranostics. 2026 Jul 29;16(14):8361–84. doi: 10.7150/thno.137729 (PMC13440567; doi:10.7150/thno.137729)
Supplement: Supplementary file 1 [file thnov16p8361s1.pdf]

Supplementary Information

A mechanobiology-driven cell-derived ECM bioink for engineering 3D glioblastoma tumor microenvironment models

Seohyeon An[1,†], *Seoyul Jo*[1,†], *GeunHyung Kim*[1,2,3,*]

[1]Department of Precision Medicine, Sungkyunkwan University School of Medicine (SKKU-SOM), Suwon 16419, Republic of Korea

[2]Institute of Quantum Biophysics, Department of Biophysics, Sungkyunkwan University, Suwon, Gyeonggi-do 16419, Republic of Korea

[3]Biomedical Institute for Convergence at SKKU (BICS), Sungkyunkwan University, Suwon 16419, Republic of Korea

[†]These authors contributed equally.

[*]Corresponding author, Prof. GeunHyung Kim: gkimbme@skku.edu

Supplementary Table

Supplementary Table S1. Gene primer sequences utilized in the RT-qPCR analyses.

Gene	Source	Primer sequence	
		Left (5'-3')	Right (3'-5')
GAPDH	Homo sapiens	GTCTCCTCTGACTTCAACAGCG	ACCACCCTGTTGCTGTAGCCAA
Piezo1	Homo sapiens	CCTGGAGAAGACTGACGGCTAC	ATGCTCCTTGGATGGTGAGTCC
YAP	Homo sapiens	TGTCCCAGATGAACGTCACAGC	TGGTGGCTGTTTCACTGGAGCA
TAZ	Homo sapiens	GAGGACTTCCTCAGCAATGTGG	CGTTTGTTCCTGGAAGACAGTCA
GDF15	Homo sapiens	CAACCAGAGCTGGGAAGATTCG	CCCGAGAGATACGCAGGTGCA
MMP2	Homo sapiens	AGCGAGTGGATGCCGCCTTTAA	CATTCCAGGCATCTGCGATGAG
MMP9	Homo sapiens	GCCACTACTGTGCCTTTGAGTC	CCCTCAGAGAATCGCCAGTACT
RUNX1	Homo sapiens	CCACCTACCACAGAGCCATCAA	TTCACTGAGCCGCTCGGAAAAG
Ki67	Homo sapiens	GAAAGAGTGGCAACCTGCCTTC	GCACCAAGTTTTACTACATCTGCC
PCNA	Homo sapiens	CAAGTAATGTCGATAAAGAGGAGG	GTGTCACCGTTGAAGAGAGTGG
CyclinD1	Homo sapiens	TCTACACCGACAACTCCATCCG	TCTGGCATTTTGGAGAGGAAGTG
CCN2	Homo sapiens	CTTGCGAAGCTGACCTGGAAGA	CCGTCGGTACATACTCCACAGA
COL4A1	Homo sapiens	TGTTGACGGCTTACCTGGAGAC	GGTAGACCAACTCCAGGCTCTC
TRPC6	Homo sapiens	GCAGACAATGGCGGTCAAGTTC	AATGGTGAAGGAGGCTGCGTGT
RHOA	Homo sapiens	TCTGTCCCAACGTGCCCATCAT	CTGCCTTCTTCAGGTTTCACCG
LOX	Homo sapiens	GATACGGCACTGGCTACTTCCA	GCCAGACAGTTTTCCTCCGCC
TNC	Homo sapiens	ATGTCCTCCTGACAGCCGAGAA	AGTCACGGTGAGGTTTTCCAGC
STAT3	Homo sapiens	CTTTGAGACCGAGGTGTATCACC	GGTCAGCATGTTGTACCACAGG
HSP70	Homo sapiens	ACCTTCGACGTGTCCATCCTGA	TCCTCCACGAAGTGGTTCACCA
MET	Homo sapiens	TGCACAGTTGGTCCTGCCATGA	CAGCCATAGGACCGTATTTCGG
ROCK1	Homo sapiens	GAAACAGTGTTCCATGCTAGACG	GCCGCTTATTTGATTCCTGCTCC
ROCK2	Homo sapiens	TGCGGTCACAACTCCAAGCCTT	CGTACAGGCAATGAAAGCCATCC
EGFR	Homo sapiens	AACACCCTGGTCTGGAAGTACG	TCGTTGGACAGCCTTCAAGACC
HIF-1a	Homo sapiens	TATGAGCCAGAAGAACTTTTAGGC	CACCTCTTTTGGCAAGCATCCTG
VEGFA	Homo sapiens	TTGCCTTGCTGCTCTACCTCCA	GATGGCAGTAGCTGCGCTGATA
TWIST1	Homo sapiens	GCCAGGTACATCGACTTCCTCT	TCCATCCTCCAGACCGAGAAGG
HGF	Homo sapiens	GAGAGTTGGGTTCTTACTGCACG	CTCATCTCCTCTTCCGTGGACA
TGF-b	Homo sapiens	TACCTGAACCCGTGTTGCTCTC	GTTGCTGAGGTATCGCCAGGAA
IL-6	Homo sapiens	AGACAGCCACTCACCTCTTCAG	TTCTGCCAGTGCCTCTTTGCTG
PDGF- b	Homo sapiens	GAGATGCTGAGTGACCACTCGA	GTCATGTTCAGGTCCAACTCGG
CXCL12	Homo sapiens	CTCAACACTCCAAACTGTGCCC	CTCCAGGTACTCCTGAATCCAC
COL1A1	Homo sapiens	GATTCCCTGGACCTAAAGGTGC	AGCCTCTCCATCTTTGCCAGCA
FN1	Homo sapiens	ACAACACCGAGGTGACTGAGAC	GGACACAACGATGCTTCCTGAG
a -SMA	Homo sapiens	CTATGCCTCTGGACGCACAACT	CAGATCCAGACGCATGATGGCA
S100A4	Homo sapiens	CAGAACTAAAGGAGCTGCTGACC	CTTGGAAGTCCACCTCGTTGTC
FAP	Homo sapiens	GGAAGTGCCTGTTCCAGCAATG	TGTCTGCCAGTCTTCCCTGAAG
PDPN	Homo sapiens	GTGCCGAAGATGATGTGGTGAC	GGACTGTGCTTTCTGAAGTTGGC
EVA1B	Homo sapiens	TGCTCGTCATCAGCATCTCGTG	CAGCCGAGTCACCGTGTCCTC
DDIT4	Homo sapiens	GTTTGACCGCTCCACGAGCCT	GCACACAAGTGTTCATCCTCAGG
CD31	Homo sapiens	AAGTGGAGTCCAGCCGCATATC	ATGGAGCAGGACAGGTTCAGTC
VE-cadherin	Homo sapiens	GAAGCCTCTGATTGGCACAGTG	TTTTGTGACTCGGAAGAACTGGC
KDR	Homo sapiens	GGAACCTCACTATCCGCAGAGT	CCAAGTTCGTCTTTTCCTGGGC
APLN	Homo sapiens	CCAGAGGGTCAAGGAATGGGC	ATAACCGCCGGGGGTGGGCA
ANGPT2	Homo sapiens	ATTCAGCGACGTGAGGATGGCA	GCACATAGCGTTGCTGATTAGTC
ESM1	Homo sapiens	ACTTGCTACCGCACAGTCTCAG	AATCCATCCCGAAGGTGCCGTA
PLVAP	Homo sapiens	CAATCAGAGGTACATGGCTGCC	CTATCTCCACCTCCAGCGTCTT
CD133	Homo sapiens	CACTACCAAGGACAAGGCGTTC	CAACGCCTCTTTGGTCTCCTTG
CD44	Homo sapiens	CCAGAAGGAACAGTGGTTTGGC	ACTGTCCTCTGGGCTTGGTGTT

Vimentin	Homo sapiens	AGGCAAAGCAGGAGTCCACTGA	ATCTGGCGTTCCAGGGACTCAT
MGMT	Homo sapiens	CCTGGCTGAATGCCTATTTCCAC	GCAGCTTCCATAACACCTGTCTG
ABCG2	Homo sapiens	GTTCTCAGCAGCTCTTCGGCTT	TCCTCCAGACACACCACGGATA
P53	Homo sapiens	CCTCAGCATCTTATCCGAGTGG	TGGATGGTGGTACAGTCAGAGC

Supplementary Figures

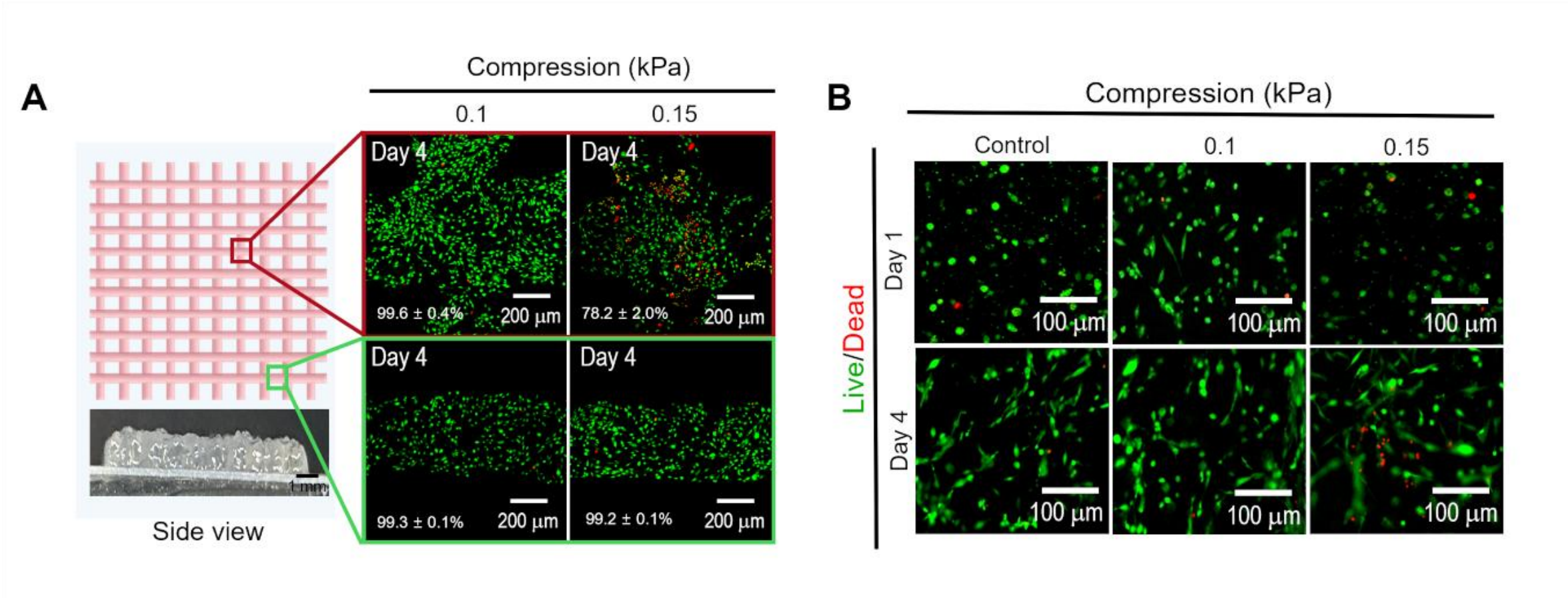

Figure S1. (A) Schematic of the bioprinted mesh construct and representative Live/Dead images and cell viability (n = 3) obtained from the indicated regions after 4 days of culture under 0.1 and 0.15 kPa compression. **(B)** Representative Live/Dead images of 3D bulk cell-laden hydrogel constructs cultured under non-compressed control, 0.1 kPa, and 0.15 kPa compression conditions at days 1 and 4.

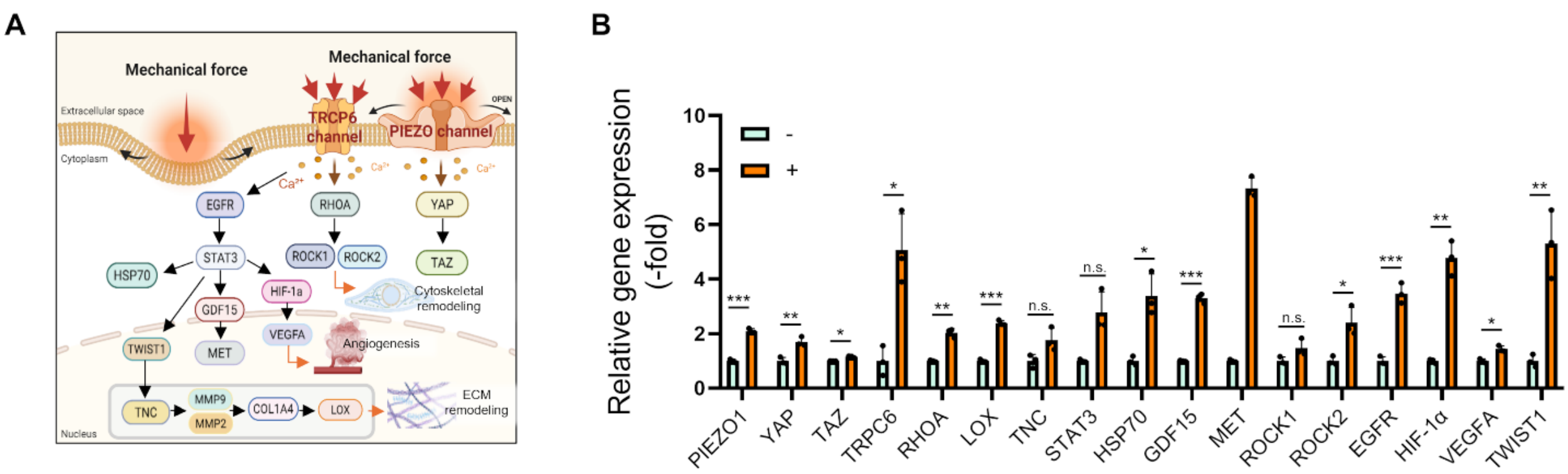

Figure S2. (A) Mechanotransductive signaling cascade linking mechanical force to pro-tumorigenic remodeling in the GBM microenvironment. **(B)** Comparative quantitative analysis of gene expression levels between non-compression (-) and compression (+) (n = 3). Student's t-test was used for two-group comparisons.

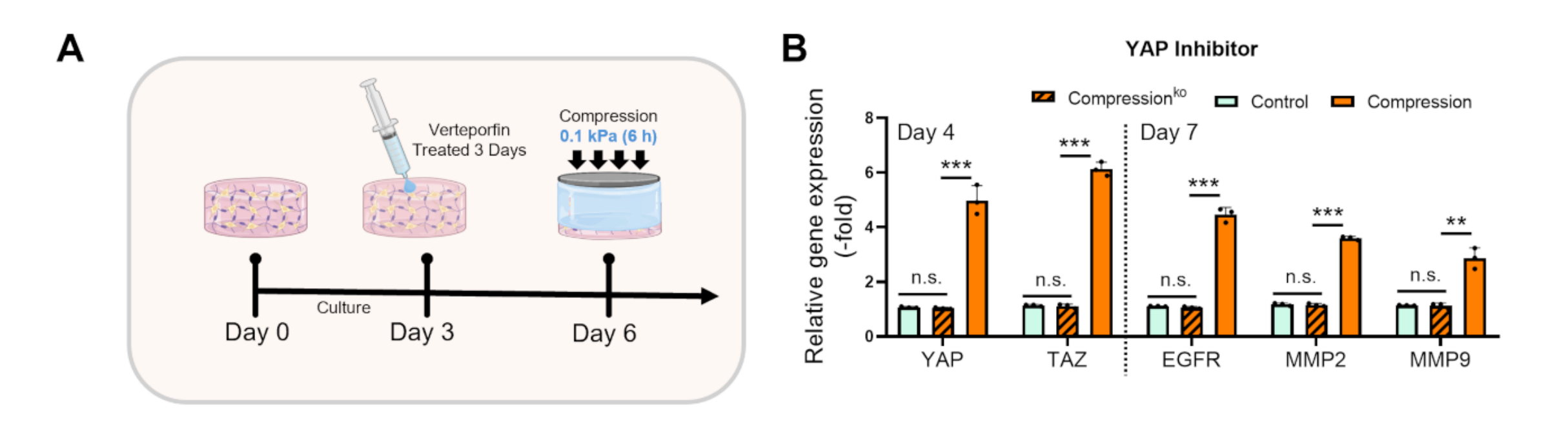

Figure S3. (A) Experimental timeline of the verteporfin treatment assay. **(B)** Relative expression (n = 3) of YAP and TAZ on day 6 and the downstream tumor progression- and ECM remodeling-associated genes (*EGFR, MMP2*, and *MMP9*) on day 9 in the control (Control), verteporfin-treated compression (Compression^{ko}), and untreated compression groups (Compression). One-way ANOVA followed by Tukey's HSD post-hoc test was used for multiple comparisons.

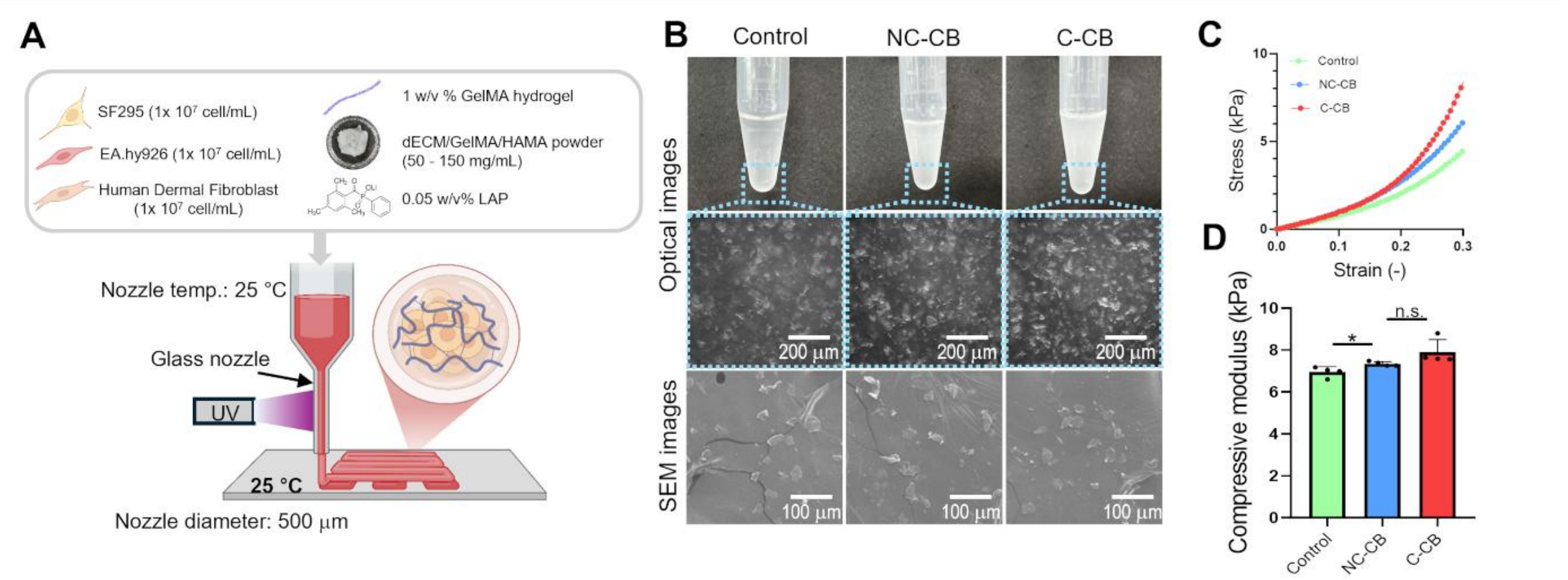

Figure S4. (A) Schematic illustration of the bioink preparation and extrusion-based bioprinting process. **(B)** Representative gross, optical, and SEM images of the control, NC-CB, and C-CB constructs, showing comparable overall morphology and surface microstructure among the groups. **(C,D)** Representative compressive stress–strain curves and corresponding compressive moduli of the control, NC-CB, and C-CB constructs (n = 4). One-way ANOVA followed by Tukey's HSD post-hoc test was used for multiple comparisons.

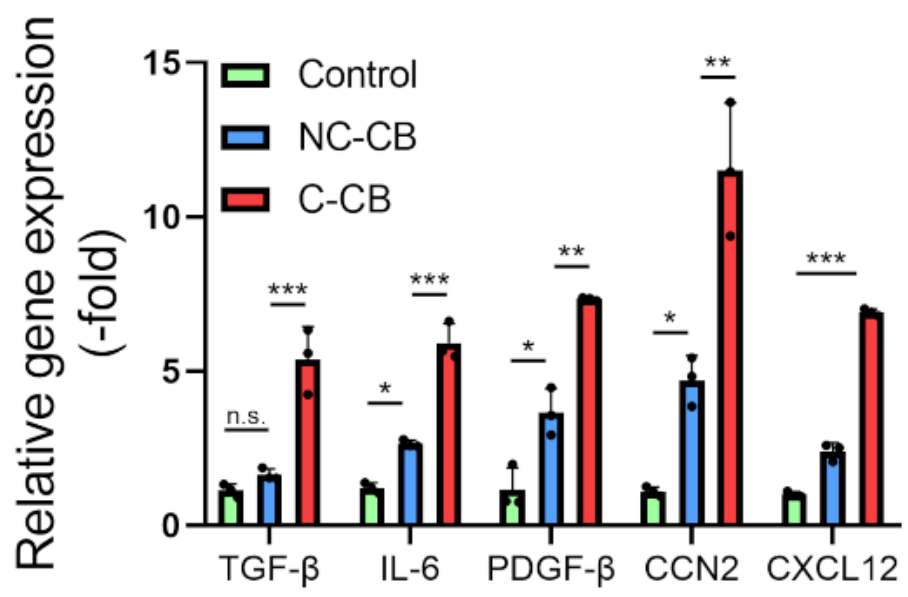

Figure S5. Comparative quantitative analysis of gene expression levels for the cell-constructs (n = 3). One-way ANOVA followed by Tukey's HSD post-hoc test was used for multiple comparisons.

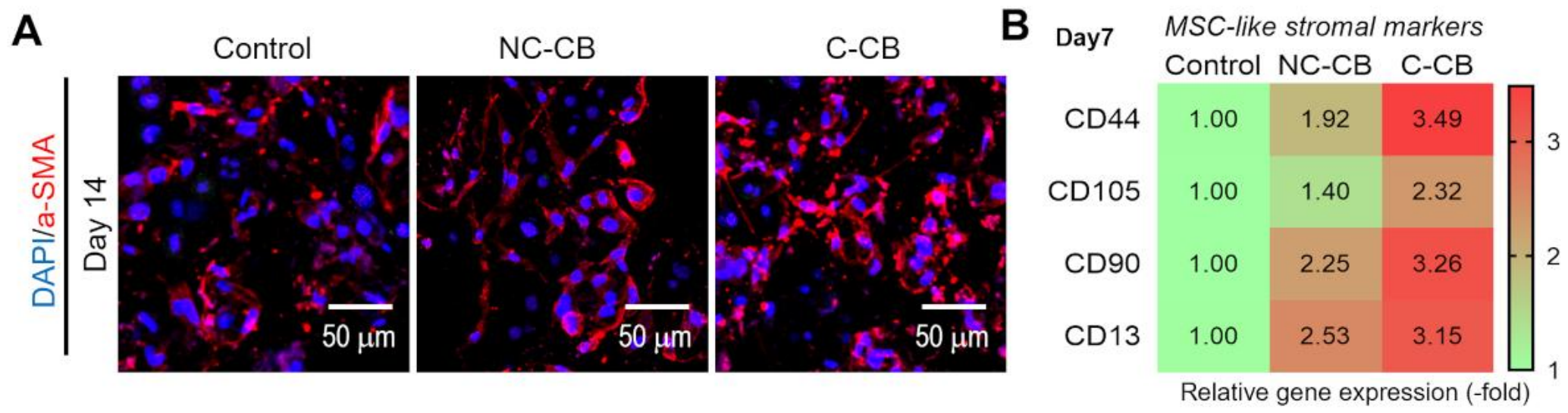

Figure S6. (A) DAPI (blue)/α-SMA (red) immunofluorescence images of the control, NC-CB, and C-CB constructs**. (B)** Heatmap of mesenchymal and tumor-associated endothelial marker expression in GBM–EA co-culture constructs (n = 3).

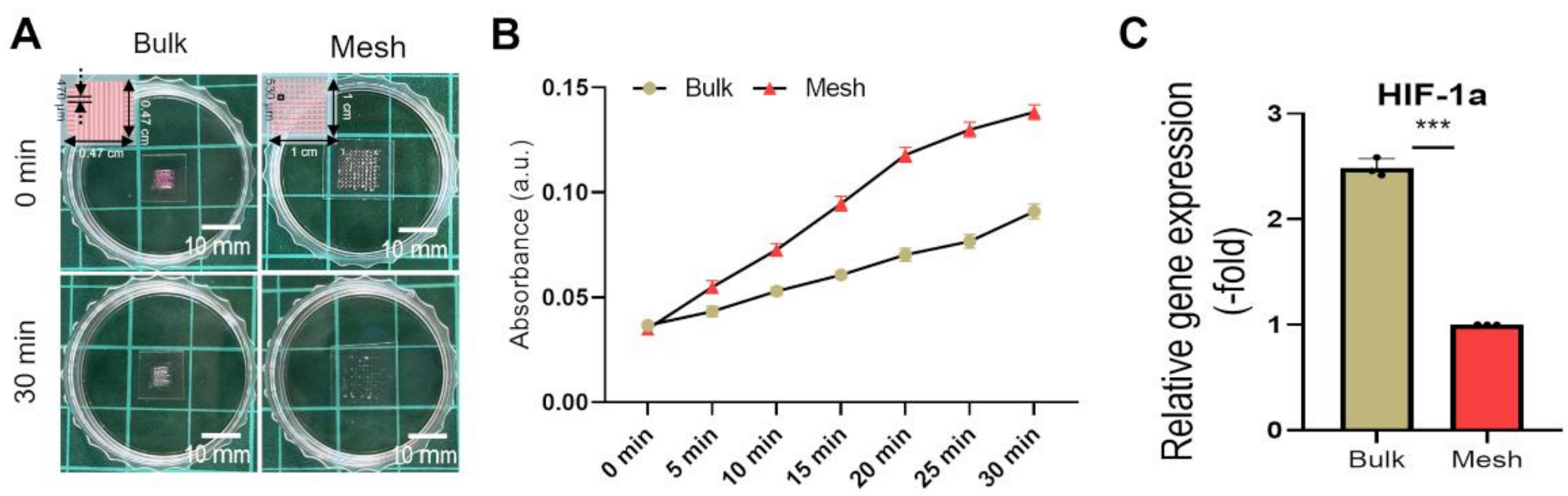

Figure S7. (A) Optical images of bulk and mesh constructs at 0 and 30 min after immersion in the PBS. Rhodamine B was incorporated into the GelMA-based bioink before photocrosslinking. **(B)** Time-dependent absorbance of rhodamine B released into the surrounding solution (n = 3). **(C)** Relative gene expression (n = 3) of *HIF-1α* of bulk and mesh biocontructs. Student's t-test was used for two-group comparisons.

Figure 1G (GAPDH, Piezo1, YAP, GDF15, MMP2, MMP9)

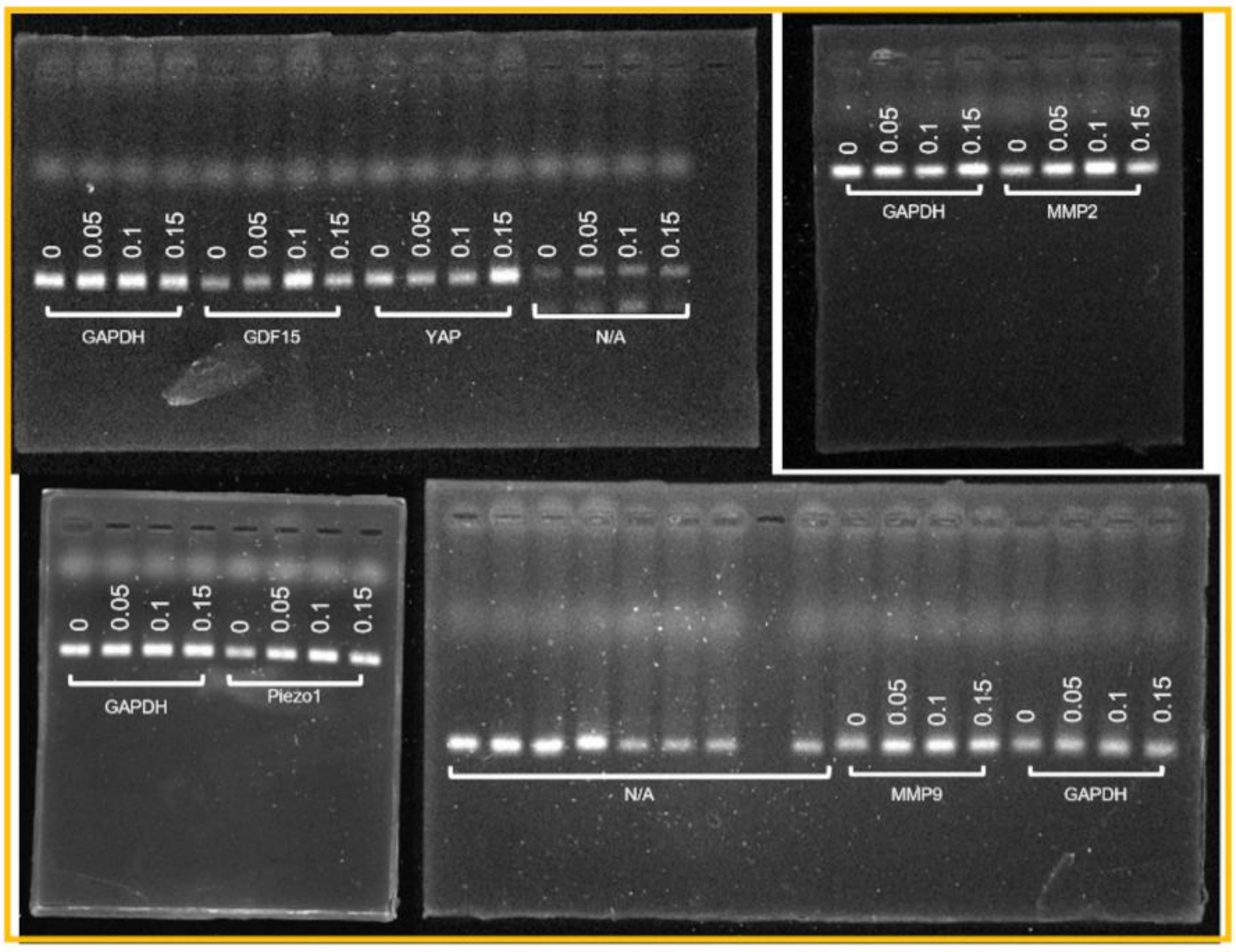

Figure 3F (GAPDH, RHOA,LOX,EGFR,HIF-1α, STAT3, MET, TWIST1, TNC, GDF15)

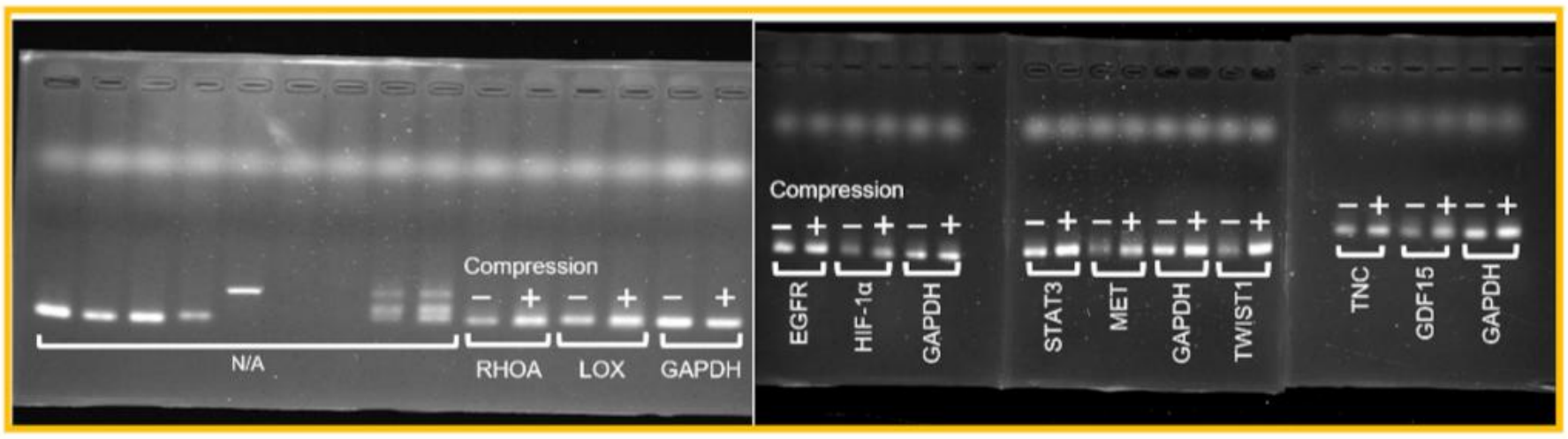

Figure 3K (GAPDH, Piezo1, EGFR, MMP2,MMP9)

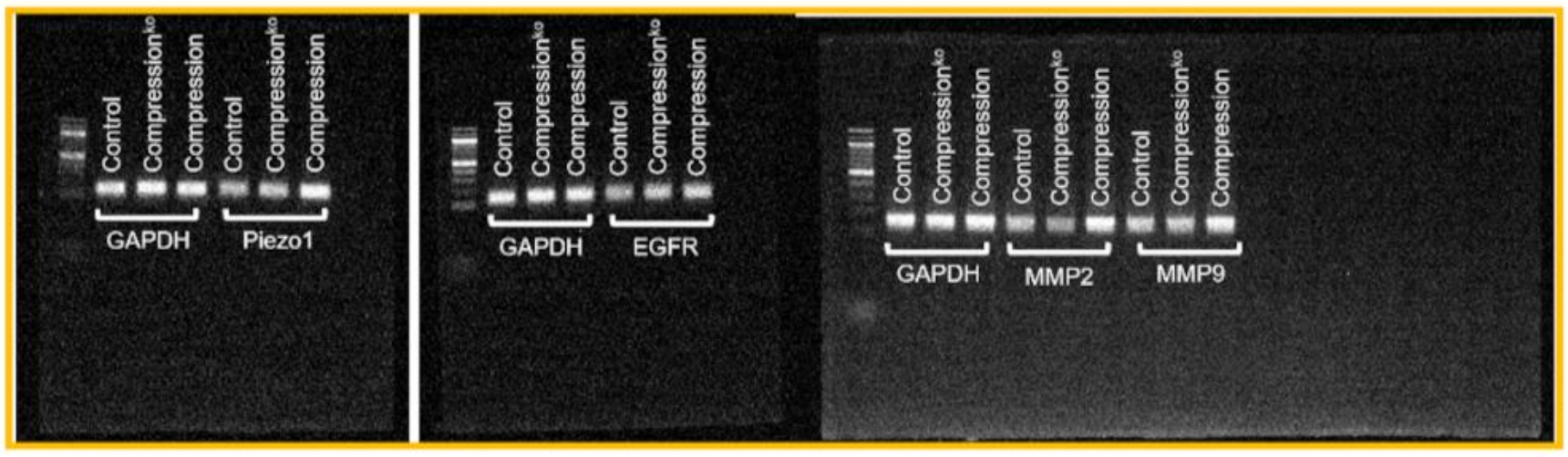

Figure 6G (GAPDH, PDGF-β, IL-6, CD133, EGFR)

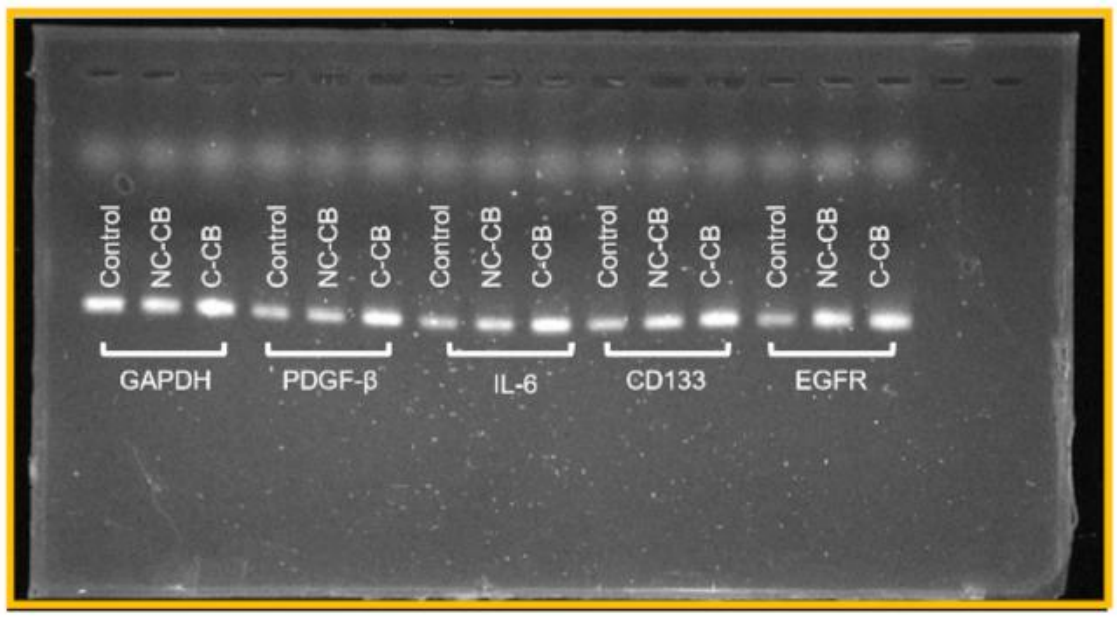

Figure 7G Day3 (GAPDH, COL1A1, α-SMA, SI00A4, PDPN)

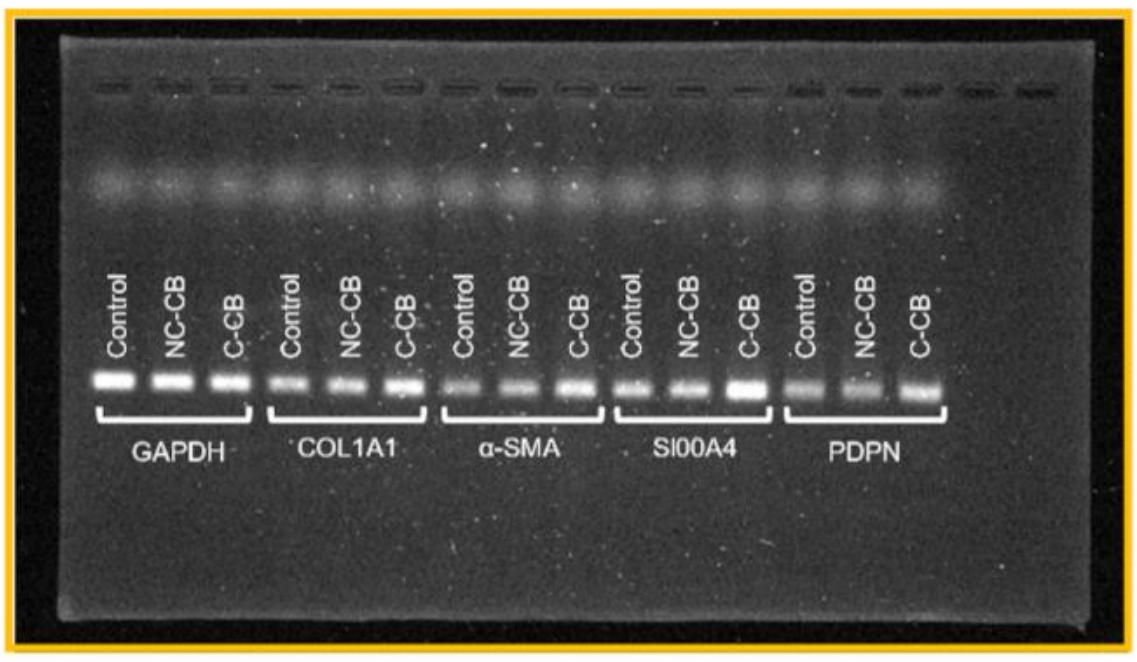

Figure 7G Day10 (GAPDH, COL1A1, α-SMA, SI00A4, PDPN)

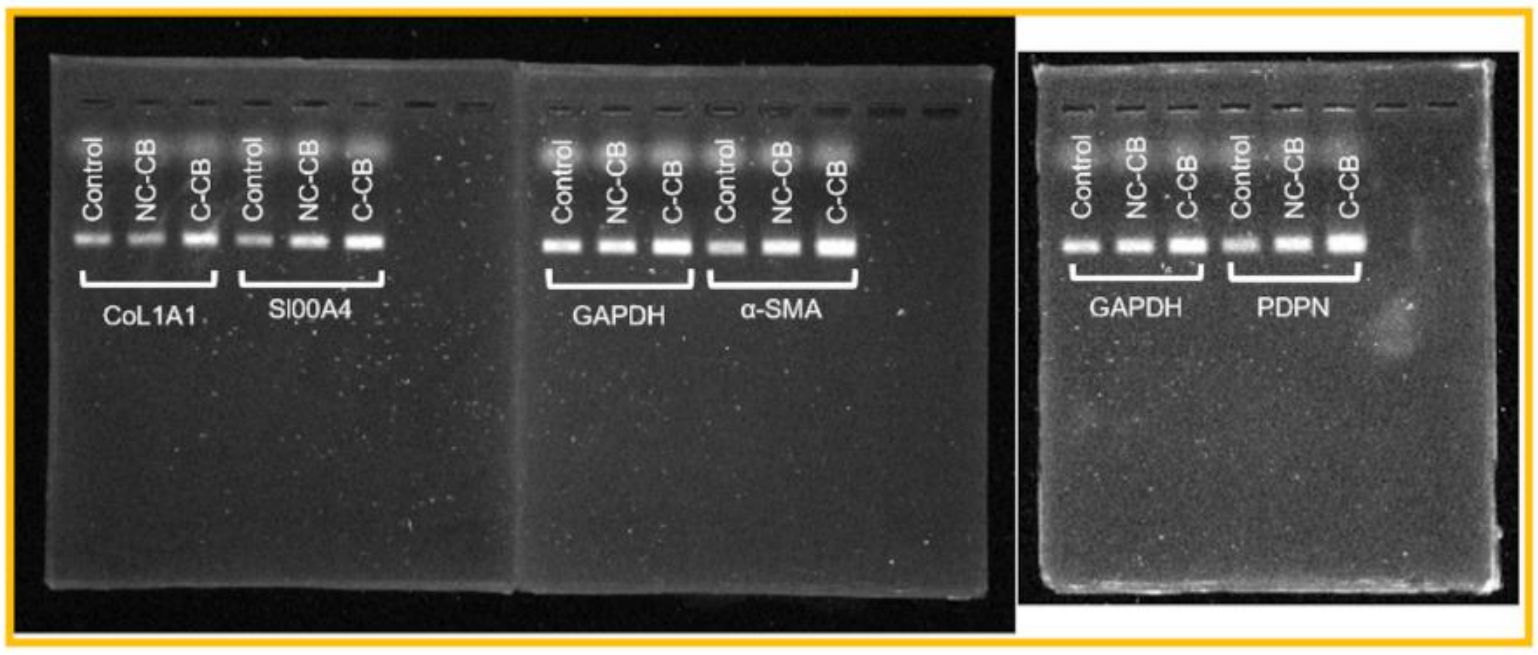

Figure 8F Day7 (GAPDH, CD31, α-SMA)

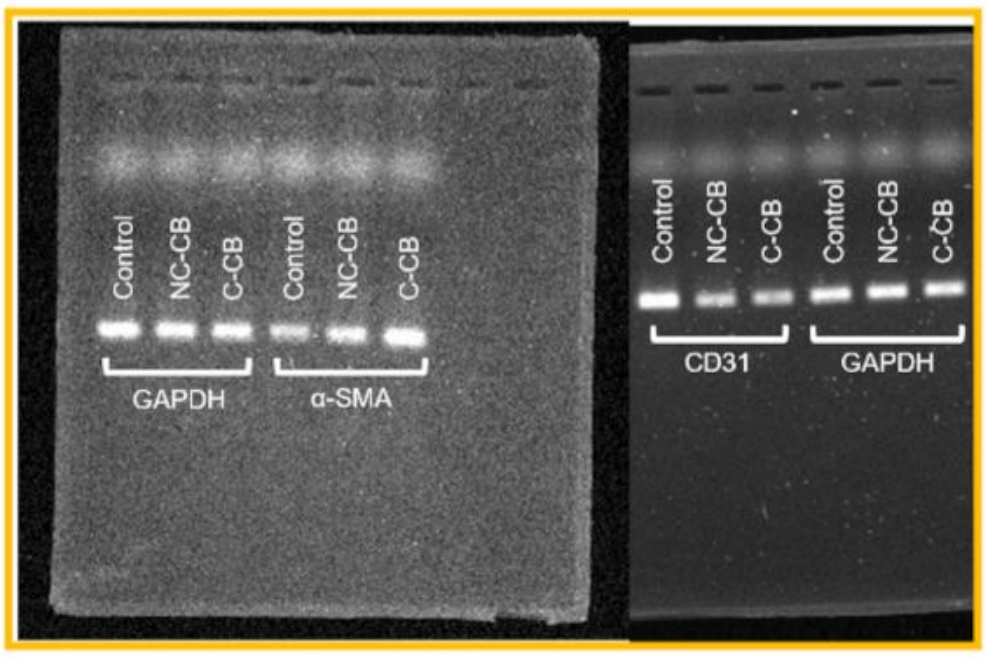

Figure 8F Day14 (GAPDH, CD31, α-SMA)

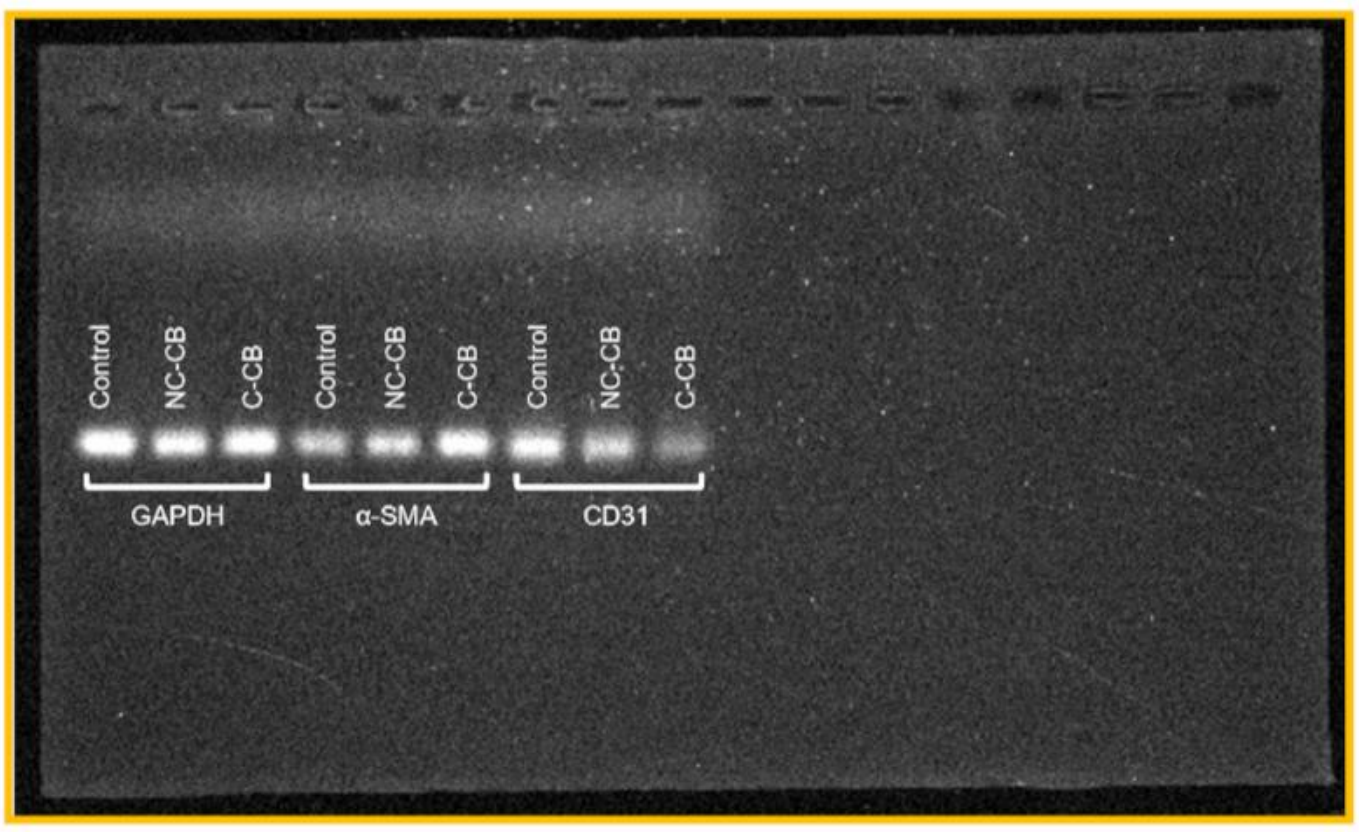

Figure S8. Raw gel electrophoresis images for each figure.
